# Genetic basis controlling rice plant architecture and its modification for breeding

**DOI:** 10.1270/jsbbs.22088

**Published:** 2023-03-29

**Authors:** Wakana Tanaka, Takaki Yamauchi, Katsutoshi Tsuda

**Affiliations:** 1 Graduate School of Integrated Sciences for Life, Hiroshima University, 1-4-4 Kagamiyama, Higashi-Hiroshima, Hiroshima 739-8528, Japan; 2 Bioscience and Biotechnology Center, Nagoya University, Furo-cho, Chikusa, Nagoya, Aichi 464-8601, Japan; 3 National Institute of Genetics, 1111 Yata, Mishima, Shizuoka 411-8540, Japan; 4 Department of Genetics, School of Life Science, Graduate University for Advanced Studies, 1111 Yata, Mishima, Shizuoka 411-8540, Japan

**Keywords:** shoot, root, leaf, stem, inflorescence, growth and development

## Abstract

The shoot and root system architectures are fundamental for crop productivity. During the history of artificial selection of domestication and post-domestication breeding, the architecture of rice has significantly changed from its wild ancestor to fulfil requirements in agriculture. We review the recent studies on developmental biology in rice by focusing on components determining rice plant architecture; shoot meristems, leaves, tillers, stems, inflorescences and roots. We also highlight natural variations that affected these structures and were utilized in cultivars. Importantly, many core regulators identified from developmental mutants have been utilized in breeding as weak alleles moderately affecting these architectures. Given a surge of functional genomics and genome editing, the genetic mechanisms underlying the rice plant architecture discussed here will provide a theoretical basis to push breeding further forward not only in rice but also in other crops and their wild relatives.

## Introduction

Rice is one of the most important staple foods in the world which supports more than 20% of human calorie consumption (Khush 2003). Several features of research infrastructures, such as a high-quality reference genome (IRGSP and Sasaki 2005, Kawahara *et al.* 2013), the feasibility of transformation (Hiei *et al.* 1994, Hiei and Komari 2008, Shimizu-Sato *et al.* 2020, Toki *et al.* 2006), and rich resources of genetic materials including mutants, cultivars and wild relatives (Sato *et al.* 2021), place rice as a model crop in plant biology.

Plant architecture, which is determined by the size, number and relative position of each organ, is the primary component affecting crop productivity. Domesticated crop species generally show similar modifications in traits including plant architecture from their wild ancestor and this phenomenon is called “domestication syndrome” (Meyer and Purugganan 2013). In rice, there are two cultivated species, *Oryza sativa* and *O. glaberrima*. Domestication of *O. sativa* from its wild ancestor *O. rufipogon* occurred in Asia approximately 10,000 years ago, whereas *O. glaberrima* was independently domesticated in West Africa from *O. barthii* about 3000 years ago (Linares 2002, Sweeney and McCouch 2007). Despite their independent domestication events, both Asian and African rice share a typical shift from their ancestors such as loss of seed shattering and prostrate to erect shoot habit (Jin *et al.* 2008, Konishi *et al.* 2006, Li *et al.* 2006, Tan *et al.* 2008, Wang *et al.* 2014, Wu *et al.* 2018). Post-domestication selection to achieve yield gain led to further morphological changes including semi-dwarfism and increased inflorescence branching (Wing *et al.* 2018). Understanding the genetic and molecular mechanisms of growth and development provides a theoretical basis for modifying plant architecture and thus, is fundamental for crop improvement and de novo domestication of wild relatives and orphan crops.

Monocotyledonous plants, including grasses, diverged from the eudicot sister clade early in angiosperm evolution and account for 25% of extant angiosperm diversity (Christenhusz and Byng 2016). Morphologies typical in grasses such as a single cotyledon, parallel venation, fibrous roots and sheathing leaves are defining features of monocots (Chase 2004). Studying the molecular mechanisms regulating these characters potentially provides insights into monocot evolution, adding further importance to grass developmental biology.

The small genome size (373.2 Mb) enabled rice to be the first crop whose high-quality genome was resolved (IRGSP and Sasaki 2005). Since then, many studies identified key regulators and QTLs for rice morphogenesis and architecture (see this review). The recent development of type II clustered regularly interspaced short palindromic repeat (CRISPR)-associated endonuclease 9 (CRISPR/Cas9) genome editing further accelerated studies of gene function (Endo *et al.* 2015, 2016, Jinek *et al.* 2012, Mikami *et al.* 2015). In addition, the emergence of high-throughput DNA sequencing technologies in the last two decades resulted in a surge in rice genomics, and these large-scale studies using cultivars and wild relatives have shed light on the processes of rice domestication and post-domestication breeding (Chen *et al.* 2019, Wing *et al.* 2018). Combined with advanced CRISPR/Cas9 genome editing techniques, de novo domestication of wild relatives and orphan crops has become recognized as a novel approach to crop breeding (Khan *et al.* 2019, Varshney *et al.* 2012, Yu *et al.* 2021).

Here, we review the current understanding of the developmental mechanisms in rice, especially by focusing on the processes that affect plant architecture. Identification of regulatory factors from developmental mutants revealed that rice shares common mechanisms with other eudicots but also has grass- or monocot-specific aspects. By referring to trait transitions involved in rice domestication and post-domestication, we see that modifications at many loci of developmental regulators had been indeed involved in the trait improvements. Through discussions with these themes, we aim to provide the developmental framework of rice plant architecture and potential avenues for future breeding.

## Meristems in rice

Meristems are the keystone for continuous organ formation during plant development. The meristems can be divided into two types: one that forms during embryogenesis, and the other that emerges at certain growth stages during post-embryonic development. The former includes the aboveground shoot apical meristem (SAM), which differentiates leaves and stems and supports the growth of the primary shoot, and the underground root apical meristem (RAM), which is responsible for the development of the seminal root (in this review, we refer to rice primary root as the “seminal root”). The latter, on the other hand, includes axillary meristems, which grow into tillers in the vegetative phase and inflorescence branches in the reproductive phase, and secondary RAMs, which produce crown roots and lateral roots.

**Fig. 1.**
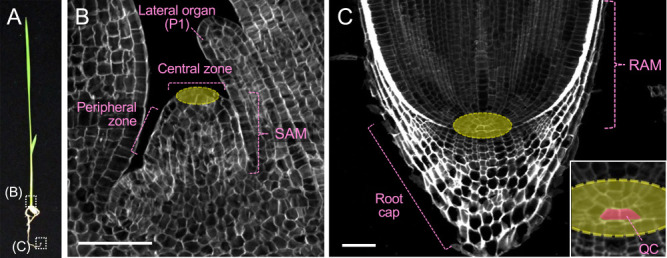
Structure of shoot and root apical meristems in rice. (A) A rice seedling (Taichung65) one week after germination. Locations of shoot and root apical meristems detailed in (B) and (C) are shown with dotted boxes. (B) Confocal micrograph of the shoot apical meristem (SAM). The putative stem cell region is indicated in yellow color. (C) Confocal micrograph of the root apical meristem (RAM). The putative initial cell region is indicated in yellow color. The inset is an enlarged view of the quiescent center (QC). The exact number of QC cells is controversial. Bars = 50 μm.

The shoot meristems consist of approximately 100 to 300 undifferentiated cells depending on the stage of leaf primordium initiation in rice (Nosaka-Takahashi *et al.* 2022). They have two main functional zones: a central zone, which is composed of slowly dividing cells, and a peripheral zone, which is composed of rapidly dividing cells and differentiates lateral organs (Fig. 1A, 1B) (Leyser and Day 2002). At the tip of the central zone, pluripotent stem cells are present. The stem cells maintain an almost constant number of themselves through self-renewal, while at the same time supplying cells to the peripheral zone. Once the fate of the cells supplied to the peripheral zone is determined, a primordium of the lateral organ such as a leaf and floral organ differentiates.

The RAMs are not located at the most apical part of the root, but lie just behind the root cap, which consists of the columella and the lateral cap (Fig. 1A, 1C) (Leyser and Day 2002). In the RAMs, stem cells (also called “initial cells”) are arranged around a small group of cells, called the “quiescent center (QC)”, although the exact number of the QC cells in rice roots is still controversial (see the subsection “*Mechanism of radial patterning of crown root and its maintenance*”) (Ni *et al.* 2014, Strotmann and Stahl 2021). The QC cells themselves usually do not divide but have an important role to promote the proliferation of the surrounding stem cells. Cells supplied from stem cells undergo active cell divisions and give rise to multiple tissues that make up the root (Details are described in the section “Root”).

The structures of both the shoot meristems and the RAMs in rice are basically similar to those in *Arabidopsis* with some exceptions. For example, the RAM of the rice seminal root appears more laterally spread out than the RAM of the *Arabidopsis* primary root, because the rice root has multiple cortex layers while the *Arabidopsis* root possesses only a single cortex layer. In the following section, we introduce the genetic basis of shoot meristem function. We will come back to the RAM in the section “Root”.

## Shoot meristems

### Maintenance of the shoot meristem indeterminacy

**Fig. 2.**
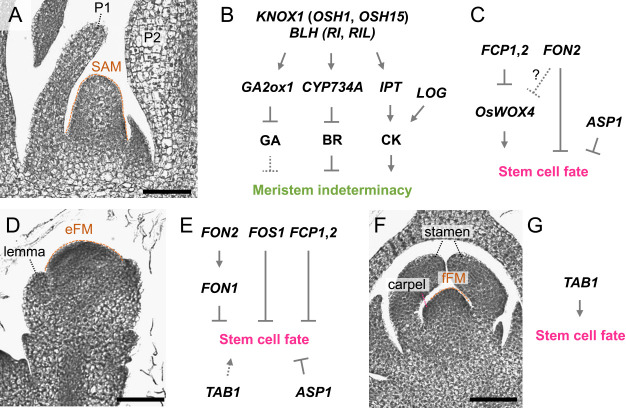
Genetic model of meristem regulation. (A) Longitudinal section of the shoot apex. A dotted orange line indicates the shoot apical meristem (SAM). (B) Model of genetic interactions involved in the maintenance of SAM indeterminacy. (C) Model of genetic interactions involved in stem cell maintenance in the SAM. (D) Longitudinal section of a flower primordium at the early stage of its development. A dotted orange line indicates the early flower meristem (eFM). (E) Model of stem cell maintenance in the eFM. (F) Longitudinal section of a flower primordium at the late stage of its development. A dotted orange line indicates the final flower meristem (fFM). (G) Model of stem cell maintenance in the fFM. Dotted arrows and T-lines represent presumptive controls that have not yet been tested in rice. P1, plastochron one leaf primordium. Bars = 50 μm.

Cells in the shoot meristems are maintained in an indeterminate state by the class I *Knotted1-like homeobox* (class I *KNOX* or *KNOX1*) genes. *knotted1* (*kn1*) in maize was the first homeobox gene cloned in plants (Vollbrecht *et al.* 1991). It is specifically expressed in shoot meristems and young stems but is down-regulated at the flank of the SAM where lateral organs differentiate (Jackson *et al.* 1994). Their gain-of-function mutants with ectopic expression in leaves or overexpressors show abnormal leaf development with retarded cell differentiation along the proximal-distal axis, therefore, *KNOX* genes are considered to maintain the shoot meristem indeterminacy by repressing cell differentiation (Chuck *et al.* 1996, Foster *et al.* 1999, Hake and Freeling 1986, Muehlbauer *et al.* 1997, 1999, Schneeberger *et al.* 1995, Sentoku *et al.* 2000, Sinha *et al.* 1993). On the other hand, loss-of-function mutants of *KNOX* genes fail to form or maintain the SAM in multiple species, indicating an indispensable role to maintain the shoot meristems (Barton and Poethig 1993, Long *et al.* 1996, Tsuda *et al.* 2011, Vollbrecht *et al.* 2000). Rice *ORYZA SATIVA HOMEOBOX1* (*OSH1*), an ortholog of maize *kn1*, is similarly expressed in the presumptive region of the SAM during embryogenesis and in the established SAM during postembryonic development (Sato *et al.* 1996, Sentoku *et al.* 1999). A loss-of-function mutant of *OSH1* possesses a slightly smaller SAM than the wild type in its embryo and terminates its growth soon after germination (Tsuda *et al.* 2011). A double mutant of *OSH1* and its close paralog *OSH15*, completely lacks the SAM in its embryo, indicating that the two genes act redundantly to establish the SAM in rice (Fig. 2A, 2B) (Tsuda *et al.* 2011). When regenerated from calli, *osh1* homozygous shoots can continue to grow and occasionally produces inflorescences, although these inflorescences are smaller than wild type and sterile due to incomplete floral organ formation (Tsuda *et al.* 2011). Recently, *OSH1* was shown to be required for the maintenance of undifferentiated cells also in the axillary meristem that gives rise to a tiller (Tanaka *et al.* 2019). Thus, these phenotypes imply that *OSH1* is involved in the regulation of shoot meristems throughout the life cycle.

*KNOX* genes encode homeodomain transcription factors which belong to the three amino acid loop extension (TALE) superfamily widely conserved among eukaryotes (Mukherjee *et al.* 2009). They form heterodimers with members of another TALE subfamily BELL1-like homeodomain (BLH) transcription factors (Hackbusch *et al.* 2005, Smith *et al.* 2002). Two rice *BLH* genes, *VERTICILLATE RACHIS* (*RI*) and *RI-LIKE* (*RIL*), are also essential for shoot meristem regulation, because their single and double mutants show several shoot meristem defects including abnormal inflorescence branching and shoot meristem-less phenotype in embryo, respectively (Fig. 2B) (Ikeda *et al.* 2019).

A wide variety of downstream targets have been identified for *KNOX* genes. Their primary role is to regulate hormonal levels in the shoot meristems (Tsuda and Hake 2015). KNOX proteins inhibit the accumulation of gibberellic acid (GA) and brassinosteroids (BR) through direct activation of their catabolic enzyme genes *GA2ox1* and *CYP734A* genes, respectively, or by repressing their synthesis (Bolduc and Hake 2009, Hay *et al.* 2002, Sakamoto *et al.* 2001, Tsuda *et al.* 2014). Meanwhile, they activate cytokinin (CK) synthesis through up-regulation of *ADENYLATE* *ISOPENTENYLTRANSFERASE* (*IPT*) genes to promote meristem activities (Fig. 2B) (Jasinski *et al.* 2005, Sakamoto *et al.* 2006, Yanai *et al.* 2005). They also inhibit secondary cell wall formation by repressing lignin biosynthesis genes (Mele *et al.* 2003, Yoon *et al.* 2017). Genome-wide analysis by chromatin immunoprecipitation followed by sequencing (ChIP-seq) revealed a massive number of potential targets, including hormone synthesis and signaling for auxin, GA, CK, BR and ethylene as well as a broad range of regulators involved in shoot development (Bolduc *et al.* 2012, Tsuda *et al.* 2014). This breadth of targets implies that *KNOX* genes work as a major hub in the diverse regulatory networks operating in shoot meristems. Regulatory relationships with these diverse targets, mechanisms of KNOX proteins in regulating their target genes and the involvement of BLH cofactors await further investigation.

### Genetic regulation of stem cell maintenance

Stem cells in the meristems balance self-renewal and cell supply for organogenesis to maintain themselves at an almost constant number. This balance (henceforth referred to as “stem cell maintenance”) is controlled by a coordinated action of positive and negative factors. In *Arabidopsis*, these positive and negative factors are WUSCHEL (WUS) and the CLAVATA (CLV) signaling, respectively. *WUS* encodes a homeodomain transcription factor and promotes stem cell proliferation (Laux *et al.* 1996, Mayer *et al.* 1998). By contrast, the CLV signaling, which is triggered by a small peptide CLV3, negatively regulates stem cell fate (Brand *et al.* 2000, Fletcher *et al.* 1999, Schoof *et al.* 2000). CLV3 is secreted from the stem cell region and perceived by receptor proteins such as CLV1, CLV2/CORYNE, and RECEPTOR-LIKE PROTEIN KINASE2 (Clark *et al.* 1997, Fletcher *et al.* 1999, Kinoshita *et al.* 2010, Muller *et al.* 2008). These receptors, in turn, transmit the signal to moderately repress the expression of *WUS* (Brand *et al.* 2000, Schoof *et al.* 2000). *WUS* is expressed in the organizing center underneath the stem cells but its protein moves to the stem cell region to promote the transcription of *CLV3* (Mayer *et al.* 1998, Schoof *et al.* 2000, Yadav *et al.* 2011). This negative feedback loop between CLV signaling and WUS is critical for the maintenance of stem cell homeostasis in all the shoot meristems.

Stem cell maintenance in grasses has been intensively studied in the past two decades, and the genetic regulation similar to *Arabidopsis* CLV signaling is considered to be basically conserved in maize and rice. In maize, *THICK TASSEL DWARF1* and *FASCIATED EAR2* (*FEA2*), which encode a CLV1-like LRR-receptor kinase and a CLV2-like LRR-receptor protein, respectively, restrict the proliferation of the inflorescence meristem (Bommert *et al.* 2005, Somssich *et al.* 2016, Taguchi-Shiobara *et al.* 2001). FEA2 transmits the signaling from two different CLV3-related peptides, ZmFON2-LIKE CLE PROTEIN1 (ZmFCP1) and ZmCLE7 (Je *et al.* 2016, 2018). Another LRR receptor, FEA3 has also been demonstrated to perceive the ZmFCP1 signaling to negatively control meristem maintenance (Je *et al.* 2016).

In rice, *FLORAL ORGAN NUMBER1* (*FON1*) and *FON2*, orthologs of *Arabidopsis* *CLV1* and *CLV3*, respectively, negatively regulate stem cell maintenance in the flower meristem from which lodicules and reproductive organs develop (Fig. 2D, 2E) (Suzaki *et al.* 2004, 2006). In either *fon1* or *fon2* mutant, the flower meristem enlarges due to an overproliferation of stem cells, resulting in an increase in the number of floral organs such as pistils and stamens. *FON1* is expressed in entire regions of shoot meristems throughout the life cycle, whereas *FON2* expression is limited in the stem cell regions in these shoot meristems (Suzaki *et al.* 2004, 2006). FON2 is considered to act as a ligand of FON1 because the effect of *FON2* overexpression is undetectable in the *fon1* mutant background, while its overexpression causes a failure in maintenance of the flower meristem in the wild-type background (Suzaki *et al.* 2006). Thus, FON2 negatively regulates stem cell fate via FON1, similar to the CLV3 action in *Arabidopsis*. Although the role of *FON2* in aerial meristems other than the flower meristem remained unknown for years, a recent study focusing on *ABERRANT SPIKELET AND PANICLE1* (*ASP1*), encoding an *Arabidopsis TOPLESS*-related transcriptional corepressor, advanced our understanding of its role. Genetic enhancer screening revealed that the *asp1* mutation enhanced the flower meristem phenotype of *fon2* (Suzuki *et al.* 2019a). Furthermore, in the *fon2 asp1* double mutant, the sizes of the SAM and the inflorescence meristem were increased compared to that in the wild type. These findings indicate that *FON2* acts as a negative regulator of stem cell maintenance, not only in the flower meristem but also in the inflorescence meristem and the vegetative SAM (Fig. 2C). Elucidation of the functional relationship between FON2 and ASP1 at the molecular level is an interesting issue for future studies.

Negative factors other than *FON* genes have also been identified in rice. *FON2 SPARE1* (*FOS1*), a paralog of *FON2*, acts redundantly with *FON2* to maintain stem cells in the flower meristem in wild rice species and indica rice (Fig. 2E) (Suzaki *et al.* 2009). *FOS1* is expressed in the entire region of all aerial meristems, in addition to the primordia of lateral organs such as leaves and floral organs. *FON2-LIKE CLE PROTEIN1* (*FCP1*) and *FCP2*, which are also paralogs of *FON2*, act in the SAM in the vegetative phase as a negative regulator of stem cell proliferation (Fig. 2C) (Suzaki *et al.* 2008). The two *FCP* genes are expressed in all aerial meristems and lateral organ primordia in a similar pattern as *FOS1*. Inducible silencing of both *FCP1* and *FCP2* expands the expression domain of the stem cell marker in the SAM, while *FCP1* overexpression cause termination of the meristem (Ohmori *et al.* 2013).

The role of *WUS* genes in grasses is somewhat different from that of *Arabidopsis*. In maize, two *WUS* orthologs, *ZmWUS1* and *ZmWUS2*, are present and there are conflicting reports regarding their expression patterns in the SAM. Expression analyses by *in situ* hybridization showed that *ZmWUS1* was expressed in the SAM at the seedling stage (Nardmann and Werr 2006). By contrast, expression of neither *ZmWUS1* nor *ZmWUS2* was detected in transcriptome analyses of isolated meristem tissues by laser microdissection or single-cell RNA sequencing in the SAM (Knauer *et al.* 2019, Satterlee *et al.* 2020). Apart from this, a recent study using a gain-of-function mutant of *ZmWUS1* called *Barren inflorescence 3* (*Bif3*) revealed that *ZmWUS1* is involved in the maintenance of the inflorescence meristem (Chen *et al.* 2021). The expression of *ZmWUS1* is clearly detected in the restricted region of the inflorescence meristem, similar to the organizing center in the *Arabidopsis* meristem (Chen *et al.* 2021, Je *et al.* 2016). Despite the suggested role of *ZmWUS1*, a loss-of-function mutant of *ZmWUS1* did not exhibit a significant phenotype, probably due to the presence of *ZmWUS2* (Chen *et al.* 2021).

The rice *WUS* ortholog, called *TILLERS ABSENT1* (*TAB1*; *OsWUS*) is not expressed in the SAM and so does not promote stem cell proliferation (Suzuki *et al.* 2019b, Tanaka *et al.* 2015). Instead, *WUSCHEL-related homeobox 4* (*OsWOX4*), the closest paralog of *TAB1*, acts as a positive factor in stem cell maintenance (Fig. 2C) (Ohmori *et al.* 2013). Inducible silencing of *OsWOX4* leads to termination of the SAM, because of a failure of stem cell maintenance. The expression of *OsWOX4* is detected in the SAM, in addition to young leaf primordia, and is negatively regulated by FCP1 (Fig. 2C). *Arabidopsis* *WOX4* is involved in the maintenance of the vascular cambium, but not in SAM function (Hirakawa *et al.* 2010). On the other hand, rice *OsWOX4* also plays an important role in leaf differentiation and vascular development, in addition to its role in the SAM (Ohmori *et al.* 2013, Yasui *et al.* 2018). Although the function of *WOX4* orthologs in grasses other than rice is not yet well characterized, *WOX4* may have acquired multiple functions during the evolution of grasses.

While a positive factor of stem cell maintenance in rice reproductive meristems was unknown for years, the *TAB1* gene was reported to promote stem cell fate in the final flower meristem (Fig. 2F, 2G) (Tanaka *et al.* 2021). In rice, unlike *Arabidopsis*, an ovule differentiates directly from the flower meristem (Yamaki *et al.* 2005, 2011). In the *tab1* mutant, the ovule fails to differentiate, and stem cells are lost precociously in the flower meristem (Tanaka *et al.* 2021). These observations indicate that *TAB1* promotes ovule differentiation by maintaining stem cells at the late stage of flower development. Although *TAB1* is expressed in the flower meristem not only at the late stage but also at the early stage, *tab1* showed no obvious abnormality related to the early flower meristem. Therefore, it is likely that stem cells in the early flower meristem are maintained by the redundant action of *TAB1* and an unknown gene(s). Future identification of the unknown gene(s) will deepen our understanding of stem cell regulation in the flower meristem in rice.

Apart from the flower meristem, *TAB1* plays a role in stem cell maintenance also during a very limited period in axillary meristem formation, which is described in detail in the section “Tiller” (Hirano and Tanaka 2020, Tanaka *et al.* 2015, Tanaka and Hirano 2020a).

A recent study in *Arabidopsis* reported that CLE40 activates *WUS* expression via *BARELY ANY MERISTEM1*, which encodes a CLV1-like protein (Schlegel *et al.* 2021). Unlike *CLV3*, *CLE40* is expressed in the peripheral zone of the meristem and its expression is negatively regulated by *WUS*. Thus, the antagonistic action of the CLV signaling and CLE40 signaling adjusts WUS activity to achieve stem cell homeostasis in the meristem. Whether positive signaling similar to CLE40 exists in grasses is another interesting issue to be addressed by future studies.

### Importance of cytokinin for shoot meristems

CK is one of the plant growth hormones known to be closely associated with meristem activity (Fig. 2B). The first indication of a relationship between CK and meristem function came from a study with grasses. Maize *abphyl1* (*abph1*) was isolated as a mutant in which the phyllotaxy changes from an alternate pattern to an opposite and decussate pattern (Giulini *et al.* 2004). Morphological analysis suggested that this phenotype is related to an enlargement of the SAM. Positional cloning revealed that *abph1* encodes an A-type response regulator that negatively regulates CK signaling, suggesting the involvement of CK in meristem activity. Not long after the report on *abph1*, a rice *lonely guy* (*log*) mutant, which displays a reduced number of inflorescence branches and floral organs, was isolated (Kurakawa *et al.* 2007). Histological and marker expression analyses showed that the meristem activity is not properly maintained in the *log* mutant. *LOG* encodes an enzyme that acts in the final step of CK biosynthesis to produce bioactive CKs and is expressed at the tip of the SAM (Fig. 2A). Thanks to the discovery of *LOG*, our understanding of the role of CK in meristem regulation is currently advanced. In particular, several studies using *Arabidopsis* have suggested that CK, which is produced by LOG in the outermost (L1) layer of the meristem, promotes stem cell proliferation by activating *WUS* expression (Chickarmane *et al.* 2012, Gordon *et al.* 2009, Gruel *et al.* 2016). In contrast to the *log* mutant, loss of *Grain number 1a* (*Gn1a*)/*CYTOKININ OXIDASE2* (*OsCKX2*) significantly increases the number of inflorescence branches and flowers (see the section “Inflorescence”) (Ashikari *et al.* 2005). Since *OsCKX2* encodes an enzyme responsible for the degradation of bioactive CKs, the phenotype of *osckx2* is considered to be caused by increased amounts of bioactive CKs. The function of *OSH1*, *OSH15* and *OsWOX4* is also known to be associated with cytokinin action (Ohmori *et al.* 2013, Sakamoto *et al.* 2006, Tsuda *et al.* 2011).

## Leaf

**Fig. 3.**
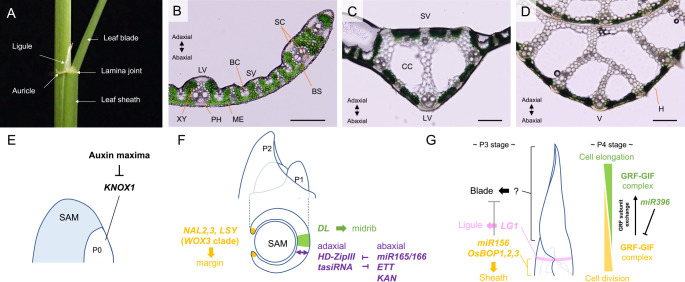
Structure of leaves in rice and regulators of its development. (A) A photograph illustrating the structure of a mature rice leaf. (B) A hand section of the marginal region of a leaf blade. (C) A hand section of the midrib. (D) A hand section of the central region of a leaf sheath. (E) The initial stage of leaf formation accompanied by KNOX down-regulation in P0. (F) Regulators along the adaxial-abaxial (purple) and medial-lateral (green and yellow) axes. (G) Regulators along the proximal-distal axis (proximal region: yellow, distal region: green, ligule: pink). Patterning processes shown on the left occur at the P3 stage, whereas the switch from cell division to cell elongation is likely to occur at P4 (right). LV: large vein, SV: small vein, BC: bulliform cell, SC: sclerenchyma, ME: mesophyll cell, XY: xylem, PH: phloem, BS: bundle sheath cell, CC: clear cell, V: vascular bundle, H: hairs. Bars = 20 μm.

As commonly seen in grasses, rice leaves are composed of the leaf sheath, the lamina joint, and the leaf blade along the proximal-distal axis (Fig. 3A) (Itoh *et al.* 2005). The leaf blade is the main site of photosynthesis and its width and thickness affect photosynthetic efficiency (Takai *et al.* 2013). The leaf sheath protects and structurally supports younger organs by enclosing them. It is advantageous to lift leaf blades for photosynthesis without stem elongation because plants can reduce the risk of damage to shoot apices by herbivore animals. The lamina joint, with appendages ligule and auricle, is a hinge-like structure to regulate leaf blade angle. Leaf blade angle is one of the important agronomical traits affecting planting density (Xu *et al.* 2021). It also relates to photosynthetic efficiency because a more upright angle allows sunlight to reach down to a larger leaf area (Xu *et al.* 2021). Thus, leaf architecture is important for both adaptive and agricultural reasons. In this section, we first describe patterning processes that determine the basic leaf shape in early development and then focus on events that elaborate mature leaf structure at later stages.

### Leaf initiation from the SAM

Leaf development proceeds with tissue patterning along adaxial-abaxial, medial-lateral and proximal-distal axes (Sylvester *et al.* 1996). Regulators involved in these processes are largely conserved among angiosperms, although there are several exceptions associated with grass-specific structures (Manuela and Xu 2020, Moon and Hake 2011). To describe leaf development, the use of “P” numbers is convenient because it unambiguously defines the developmental stages of leaves at any time point of observation. “P” stands for plastochron, a time interval between two successive leaf formations (Lamoreaux *et al.* 1978). Here, P1 is the youngest leaf primordium initiated from the SAM. The following older leaves are numbered P2, P3, and so on. The presumptive area in the SAM which is destined to be the next P1 is called P0 (Fig. 3E, 3F).

The formation of auxin maxima in the SAM is a prerequisite for initiating leaf formation (Reinhardt *et al.* 2000, 2003) (Fig. 3E). This process is regulated through the action of PIN-FORMED (PIN) auxin efflux carriers in the L1 layer of the SAM. Studies in *Arabidopsis* revealed that PIN1 proteins asymmetrically localize at the plasma membrane faced toward the position of future leaf primordia and form an auxin maximum in the L1 layer (Reinhardt *et al.* 2003). This auxin maximum coincides with P0 in which the down-regulation of class I *KNOX* genes occurs (Jackson *et al.* 1994) (Fig. 3E). A recent study in *Arabidopsis* showed that the initial down-regulation of *Arabidopsis* *KNOX* gene *STM* is mediated by the combined action of auxin response factors *ETTIN*/*ARF3* and *ARF4* and a *YABBY* transcription factor *FILLAMENTOUS* *FLOWER* (*FIL*) to recruit histone deacetylase HDA19, proposing a direct link between auxin signaling and the *KNOX* down-regulation (Chung *et al.* 2019). Thus, the initial step of leaf differentiation is a process triggered by dynamic auxin transport and loss of cell indeterminacy.

Subsequently, the PIN1 expression domain expands from the auxin maximum in the L1 layer of the SAM into inner layers down to the stem and forms a strand which will be a provascular bundle in leaves and stems (Benkova *et al.* 2003). In *Brachypodium*, the protein orthologous to *Arabidopsis* PIN1 is expressed only in these provascular bundles but not in the L1 layer (O’Connor *et al.* 2014). There is a sister clade of PIN1 proteins, called SISTER of PIN1 (SoPIN1) which is expressed in the L1 layer. SoPIN1 is essential to form the auxin maxima in the SAM and its mutation results in a naked pin-like inflorescence due to the failure of organogenesis (O’Connor *et al.* 2017). The double mutant of *SoPIN1* orthologues in rice, *pin1c pin1d*, also fails to develop inflorescence branches (Li *et al.* 2019). Importantly, this SoPIN1 clade is absent only in *Brassicaceae* including *Arabidopsis* but is present in many other angiosperm clades (O’Connor *et al.* 2014). This suggests that the auxin maxima formation in the L1 layer and the re-distribution of auxin into the inner tissue to form provascular bundles are separately regulated by distinct PIN proteins in most angiosperm species.

### Patterning along the adaxial-abaxial axis

After these events, a P1 leaf primordium initiates as a bulge from the flank of the SAM and establishes the adaxial-abaxial polarity axis (Fig. 3F). Unlike dicot leaves which have clear adaxial palisade and abaxial spongy cell layers, grass leaves including rice show a uniform configuration of mesophyll cells and symmetrically arranged sclerenchyma along vascular bundles in leaf blades (Fig. 3B) (Hoshikawa 1989). Nevertheless, rice leaves develop several features unequally distributed along the adaxial-abaxial axis; bulliform cells in the adaxial epidermis of leaf blades which cause leaf rolling upon water deficit, a ligule derived from the adaxial epidermis at the lamina joint, the smooth hairless surface on the adaxial side and abaxially localized vascular bundles in the leaf sheath (Fig. 3A, 3B, 3D) (Hoshikawa 1989). These structures should develop at the right place based on the positional cues along the adaxial-abaxial axis.

*Class III homeodomain-leucine zipper* (*HD-ZIP III*) genes encode transcription factors that promote the adaxial cell fate (Emery *et al.* 2003, Eshed *et al.* 2001, McConnell *et al.* 2001). Their expression is confined to the adaxial side by *micro RNA 165/166* (*miR165/166*) (Kidner and Martienssen 2004, Mallory *et al.* 2004). In rice, *OSHB1* to *OSHB5* belong to the *HD-ZIP III* family and are preferentially expressed in various leaf tissues on the adaxial side including the epidermal layer, bundle sheath extension cells, sclerenchyma, young ligules as well as in vascular bundles (Itoh *et al.* 2008a). Overexpression of micro-RNA resistant versions of *OSHB* genes, in which silent nucleotide substitutions were introduced into *miR165/166* target sites, results in malformed leaves accompanied by the ectopic formation of bulliform cells and ligules on the abaxial side, or in the most severe case, completely radialized filamentous leaves (Itoh *et al.* 2008a). These observations suggest that the *HD-ZIP III-miR165/166* regulatory module in adaxial cell fate specification is also conserved in rice (Fig. 3F).

Members of *KANADI* (*KAN*) family transcription factors, on the other hand, specify the abaxial cell identity (Eshed *et al.* 2004, Kerstetter *et al.* 2001). A loss of function mutant of *SHALLOT-LIKE1* (*SLL1*), a rice ortholog of *Arabidopsis* *KAN1*, displays a leaf rolling phenotype due to a lack of sclerenchyma on the adaxial side (Zhang *et al.* 2009). *sll1* mutants also show a wide range of adaxialized leaf phenotypes including the ectopic formation of bulliform cells and ligules on the abaxial side. *SLL1* is preferentially expressed in the abaxial epidermis of developing leaves, indicating that the *KAN* gene establishes the abaxial cell identity also in rice (Fig. 3F) (Zhang *et al.* 2009). *ETTIN/AUXIN RESPONSE FACTOR3* (*ARF3*) and *ARF4* are additional regulators to promote the abaxial cell fate. In *Arabidopsis*, *ett* *arf4* double mutant show adaxialized lateral organ phenotypes similar to *kan1 kan2* double mutants (Pekker *et al.* 2005). KAN and ETTIN proteins cooperatively act through a physical interaction both in *Arabidopsis* and rice (Adedze *et al.* 2018, Kelley *et al.* 2012). *ETTIN* and *ARF4* transcript accumulation is negatively regulated by adaxially generated trans-acting siRNA *TAS3* and is confined to the abaxial side (Fahlgren *et al.* 2006, Garcia *et al.* 2006, Hunter *et al.* 2006). In the ta-siRNAs biogenesis in rice, *SHOOTLESS2* (*SHL2*), *SHL4* and *SHOOT ORGANIZATION1* (*SHO1*), which encode RNA-dependent RNA polymerase, ARGONAUTE7 (OsAGO7) and DICER-like proteins, respectively, play pivotal roles (Nagasaki *et al.* 2007). Although their loss-of-function mutants were isolated as embryonic mutants that lack shoot formation, the weak allele of *SHL4* shows partially abaxialized phenotypes with expanded *OsETT* expression (Itoh *et al.* 2008b). In contrast, overexpression of *SHL4/OsAGO7* results in upward leaf rolling possibly due to adaxial-abaxial defects (Shi *et al.* 2007). Thus, these findings indicate that the adaxial-abaxial polarity in rice leaves is determined through the interplay between evolutionarily conserved transcription factors specific to each side and their regulatory small RNAs.

### Growth along with the medial-lateral axis

From the P1 to P2 stage, a leaf primordium encircles the SAM by its marginal growth along the medial-lateral axis and forms a hood-shaped primordium (Itoh *et al.* 2005) (Fig. 3F). The marginal growth is promoted by the functions of *WOX1* and *WOX3* in *Arabidopsis* (Matsumoto and Okada 2001, Nakata *et al.* 2012). In grasses, genes in the *WOX1* clade were lost (Zhang *et al.* 2010), but those in the two duplicated *WOX3* subclades play essential roles in this process. *NARROW LEAF2* (*NAL2*) and *NAL3* in rice, *NARROW SHEATH1* (*NS1*) and *NS2* in maize and a single gene *NARROW* *LEAFED DWARF1* (*NLD1*) in barley belong to one of the duplicated *WOX3* clades that are expressed at the very margin of growing leaf primordia (Cho *et al.* 2013, Ishiwata *et al.* 2013, Nardmann *et al.* 2004, Scanlon *et al.* 1996, Yoshikawa *et al.* 2016). Rice *nal2 nal3* double mutants together with corresponding loss-of-function mutants in maize and barley show narrow leaf phenotypes due to a lack of marginal growth, indicating an essential role of genes in this *WOX3* clade (Ishiwata *et al.* 2013). In another *WOX3* clade of grasses, only a rice gene *LEAF LATERALSYMMETRY1* (*LSY1*) has been functionally characterized (Honda *et al.* 2018). *LSY1* is expressed in a similar but broader region of leaf margins than *NAL2/3*, and *lsy1* mutants show irregular asymmetrical leaf phenotypes in which one or both margins were lost due to the misregulation of adaxial-abaxial identity. It is noteworthy that both of the duplicated *WOX3* clades (*NAL2/3* clade and *LSY1* clade in rice) acquired similar but non-redundant functions during grass evolution (Honda *et al.* 2018). Recently, Richardson and colleagues proposed that the *WOX3*-dependent extension of the leaf marginal zone played an important role during the establishment of the sheathing leaf trait in grasses and that the leaf sheath derives from petiole in dicot leaves (Richardson *et al.* 2021). Future studies in this area will unveil the evolutionary scenario of the leaf sheath.

The central midrib region of grass leaves has an increased thickness accompanied by two large lacuna called clear cells, a large vascular bundle on the abaxial side and an additional small vascular bundle on the adaxial side (Fig. 3C). A *YABBY* gene *DROOPING LEAF* (*DL*) promotes midrib formation in rice (Fig. 3F). In *dl* mutants, all of these characteristics of the midrib are missing in the medial region and are replaced by that of the lateral region with a single large vein (Yamaguchi *et al.* 2004). Similar defects were reported in maize *drooping leaf1* (*drl1*) *drl2* double mutants (Strable *et al.* 2017). *DL* encodes a rice ortholog of *Arabidopsis CRABS CLAW* (*CRC*). *DL* is expressed in the medial region of developing leaves, consistent with its function in midrib formation. Constitutive expression of *DL* under the rice *Actin* promoter resulted in the expansion of midrib characters into lateral regions of leaf blades (Yamaguchi *et al.* 2004). These findings show that *DL* is not merely promoting cell proliferation but rather specifying midrib identity in the medial region. *DL* is also required for carpel specification; carpels in *dl* mutants are homeotically converted to stamens. This function of *DL* is similar to that of *Arabidopsis CRC* which is involved in carpel development and floral meristem determinacy (Alvarez and Smyth 1999). However, *CRC* is not expressed in leaves (Alvarez and Smyth 1999, Bowman and Smyth 1999). Defects in carpel and midrib specification similar to *dl* mutants have been reported in two other grass species (Fladung *et al.* 1991, Rao *et al.* 1988). Therefore, *DL* function in the midrib formation is conserved at least among grasses and might have been uniquely acquired during the evolution of monocots. Taken together, the specification of the lateral laminae and the central midrib in grasses are controlled by *WOX3* genes and a *YABBY* gene *DL*, respectively. Both regulators are likely to have unique evolutionary histories and functions distinct from those in dicots. When these functional innovations occurred and how these events contributed to the evolution of monocot leaves are intriguing questions.

### Specification of structures along the proximal-distal axis

Initiation of ligule becomes visible at the P3 stage as periclinal divisions at the adaxial epidermis and separates leaf blade and sheath (Fig. 3G) (Itoh *et al.* 2005). Genetic studies in maize and rice showed that *LIGULELESS1* (*LG1*), a *SQUAMOSA PROMOTER BINDING PROTEIN-LIKE* (*SPL*) family transcription factor, is essential for ligule specification (Lee *et al.* 2007, Moreno *et al.* 1997). Interestingly, even in *lg1* mutants, leaf sheathes and blades still form in a normal ratio, indicating the presence of genetic pathways specifying these organs independent of ligule formation. Recent studies in rice identified *BLADE-ON-PETIOLE* (*OsBOP*) genes as regulators involved in this process. *OsBOP1*, *OsBOP2*, and *OsBOP3* are predominantly expressed in the proximal part of leaves and repress blade formation to promote sheath development (Fig. 3G) (Toriba *et al.* 2019). Leaves in *osbop1 osbop2 osbop3* triple mutants mostly consist of leaf blades with severely retarded sheathes. Expression levels of *OsBOP* genes are promoted by *miR156* which targets *SPL* TFs during the juvenile phase, indicating a regulatory link between leaf blade-sheath ratio and juvenile-adult phase control (Toriba *et al.* 2019). This *BOP-miR156* regulatory module also plays conserved roles in blade suppression in rhizomatous and stoloniferous species (Toriba *et al.* 2020). Thus, these studies placed *BOP* genes as regulators for the proximal part of leaves. How the fate of leaf blades is specified is an open question.

### Elaboration of the mature leaf structure

Leaf differentiation in rice proceeds basipetally (Itoh *et al.* 2005). At the P4 stage in rice, cells still actively divide at the base whereas they elongate in the distal half (Fig. 3G). GROWTH REGULATING FACTOR (GRF) family TFs activate cell proliferation together with GRF-INTERACTING FACTOR (GIF) proteins in young organs including leaves. In rice, a *GIF* gene *MAKIBA3* is expressed in the abaxial side of developing leaves and MKB3 protein moves and activates cell proliferation non-cell autonomously (Shimano *et al.* 2018). In maize, a GIF protein ANGUSTIFOLIA3 (AN3) assembles protein complexes including SNF/SWN chromatin remodelers and interacts with distinct subsets of GRFs along the proximal-distal axis (Nelissen *et al.* 2015). In the proximal region of leaf primordia, AN3 interacts with GRF1, GRF6, GRF7, GRF12, GRF15, and GRF17 to activate cell division whereas they are repressed by *miR396* in the distal regions. In the cell expansion zone of the distal half of leaves, GRF4/10, which lack *mir396* binding sites, replace these early GRFs in AN3 protein complexes and promote cell elongation (Fig. 3G).

Leaf thickness is an important factor affecting the number of mesophyll cells which contain developed chloroplasts and hence photosynthesis rate. *GREEN FOR PHOTOSYNTHESIS* (*GPS*) was identified from indica cultivars as a QTL which increases photosynthesis rate (Takai *et al.* 2013). *GPS* is a natural variation and partial loss-of-function allele of *NARROW LEAF1* (*NAL1*), which encodes a trypsin-like serine and cysteine protease. Although the molecular function of NAL1 protein in leaf development is still unknown, a recent study in rice inflorescence suggested that it modulates the abundance of a key developmental regulator at the protein level (Huang *et al.* 2018) (see the section “Inflorescence”). The *GPS* allele improves photosynthesis capacity by increasing mesophyll number and leaf thickness without detrimental effects seen in severe *nal1* mutants. This study highlights the utility of weak alleles of developmental genes for breeding.

Lamina joint inclination, or leaf blade angle, is another important trait affecting photosynthesis and planting density. A phytohormone brassinosteroid (BR) enhances the bending at lamina joints by increasing cell expansion on the adaxial side (Xu *et al.* 2021). A recent study in maize reported a QTL *UPRIGHT PLANT ARCHITECTURE2* (*UPA2*) from the ancestor teosinte (Tian *et al.* 2019). In the *UPA2* allele, there is a polymorphism in the promoter of *ZmRAVL1*, which positively regulates the expression of a BR synthesis enzyme gene *brd1*. This polymorphism causes a reduction of *ZmRAVL1* expression and hence results in the reduced expression of *brd1*. As the consequence, the growth of ligules and auricles is limited in *UPA2* plants, leading to upright leaves (Tian *et al.* 2019). Importantly, the introgression of the *UPA2* allele into maize inbreds successfully enhanced yields in dense planting conditions (Tian *et al.* 2019). Leaf blade angles can be fine-tuned in a similar way in rice and other grasses.

## Tiller

**Fig. 4.**
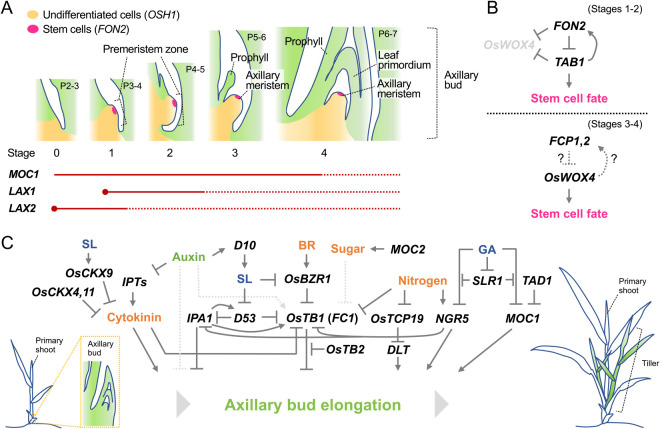
Regulatory model of axillary bud formation and elongation. (A) Schematic representation of axillary bud formation and key genes involved in this process. A solid red line next to each gene indicates the period when gene expression was observed, whereas a dotted red line indicates the period when it is not known whether the gene is expressed or not. (B) Model of stem cell regulation during axillary bud formation. A dotted arrow and T-line represent presumptive controls that have not been clarified. (C) Model of axillary bud elongation. A dotted arrow and T-line represent presumptive controls that have not yet been demonstrated in rice. Hormones and nutrients that have positive and negative effects on tillering are colored in orange and blue, respectively. The effect of auxin colored in green depends on the context/condition of the bud.

Vegetative branches that grow from the base of the primary shoot in grasses are called “tillers” (Fig. 4). Tiller formation is largely divided into two developmental processes: “axillary bud formation” and “axillary bud elongation”. In the first process, an axillary bud, which consists of an axillary meristem and surrounding leaf primordia, is formed in the leaf base at the junction of the leaf and stem, i.e., leaf axil (Fig. 4). The bud remains dormant immediately after its formation, however, when the plant receives stimuli from either the internal or external environment, the bud is released from dormancy and enters the second process, axillary bud elongation. In this process, the bud not only elongates vigorously but also grows at a certain angle to the vertical axis to form a mature tiller, which is nearly indistinguishable from the primary shoot. In this section, we focus on core factors and genetic pathways regulating tiller development and also introduce natural variations affecting tiller growth that were utilized during rice domestication and post-domestication.

### Axillary bud formation

#### Developmental processes of axillary buds

Morphological observations and spatiotemporal expression analyses of marker genes have revealed that the developmental process of axillary bud formation in rice is accompanied by dynamic morphological changes (Hirano and Tanaka 2020, Oikawa and Kyozuka 2009, Tanaka *et al.* 2015). In the axil of the P2-3 leaf of the main shoot, a very slight bulge can be observed, which is the beginning of axillary bud formation (Fig. 4A, stage 0) (Oikawa and Kyozuka 2009). In this stage, the undifferentiated cell maker *OSH1* is strongly expressed in the axil. The bulge composed of small and cytoplasm-dense cells subsequently becomes more visible in the leaf axil. In this bulge, the *OSH1* signal is hardly detectable despite the increasing number of cells (Oikawa and Kyozuka 2009). Shortly thereafter, a strong expression of *OSH1* is detected in the bulge in the axil of the P3-4 leaf (Fig. 4A, stage 1) (Oikawa and Kyozuka 2009, Tanaka *et al.* 2015). In this stage, the signal of the stem cell marker *FON2* becomes detectable in the upper region of the bulge, implying that stem cells are likely established in this stage during axillary bud formation (Tanaka and Hirano 2020a). Since the bulge in this stage is composed of undifferentiated cells and contains stem cells, it is named the “premeristem zone” (Tanaka *et al.* 2015, Tanaka and Hirano 2020a). Not until this stage, is the expression of cell division markers, *Histone H4* and *CDKB2;1*, observed in the proliferating bulge (premeristem zone) (Oikawa and Kyozuka 2009). In the axil of the P4-5 leaf, a prophyll primordium differentiates from the apical part of the premeristem zone, where the expression of *OSH1* is downregulated (Fig. 4A, stage 2) (Oikawa and Kyozuka 2009, Tanaka *et al.* 2015). In the axil of the P5-6 leaf, the prophyll becomes gradually evident and the dome of the axillary meristem is established in this stage (Fig. 4A, stage 3) (Tanaka *et al.* 2015). The *FON2* signal is detected in a few outer layers of this meristem dome, a pattern similar to that detected in the SAM (Suzaki *et al.* 2006, Tanaka and Hirano 2020a). The primordia of foliage leaves start to differentiate from the axillary meristem in the axil of the P6-7 leaf (Fig. 4A, stage 4).

#### Transcription factors essential for the initiation of axillary buds

Genetic studies have shown that several transcription factors are involved in axillary bud formation. *MONOCULM1* (*MOC1*) encoding a GRAS family transcription factor is the first gene identified in rice that regulates the number of tillers (Li *et al.* 2003). A loss-of-function mutant of *MOC1* exhibits a monoculm phenotype without tillers. Anatomical analysis of *moc1* revealed that no trace of the bud formation was observed in the leaf axil, indicating that initiation of axillary buds is impaired. *MOC1* starts to be expressed in a small number of cells in the presumptive region of the axillary bud in the P2 leaf axils (Fig. 4A, stage 0). All these data suggest that *MOC1* plays an essential role in the initiation of axillary bud formation. *LAX PANICLE1* (*LAX1*) and *LAX2*, which encode a basic helix-loop-helix transcription factor and a nuclear protein with a plant-specific conserved domain, respectively, also participate in this process (Komatsu *et al.* 2003a, Oikawa and Kyozuka 2009, Tabuchi *et al.* 2011). While both single mutants of *lax1* and *lax2* are still capable of forming tillers, their double mutant completely lacks tillers, indicating a synergistic effect of these mutations (Tabuchi *et al.* 2011). Although *LAX1* transcript is specifically detected in the boundary region at the adaxial side of the bulge (premeristem zone), LAX1 protein is trafficked and accumulates in the entire bulge (Fig. 4A) (Komatsu *et al.* 2003a, Oikawa and Kyozuka 2009). *LAX2* is also expressed in the whole region of the premeristem zone in a similar stage (Fig. 4A) (Tabuchi *et al.* 2011). Given that LAX1 and LAX2 proteins physically interact in vitro, these two proteins are likely to act cooperatively in the premeristem zone to promote axillary meristem formation (Tabuchi *et al.* 2011). The transcription factors closely related to MOC1 and LAX1 in *Arabidopsis* and tomato are required for axillary bud formation as well (Greb *et al.* 2003, Schumacher *et al.* 1999, Yang *et al.* 2012), suggesting that the molecular control of bud initiation is partly conserved between monocots and eudicots.

#### Regulators of stem cell fate in axillary bud formation

Rice *WUS* ortholog, *TAB1* (also known as *OsWUS*, *MONOCULM3*, and *COMPLETELY STERILE AND REDUCED TILLERING1*) plays an essential role in axillary bud formation (Hirano and Tanaka 2020, Lu *et al.* 2015, Mjomba *et al.* 2016, Tanaka *et al.* 2015, Tanaka and Hirano 2020a), but its role is quite different from those of transcription factors described above. Unlike *Arabidopsis wus*, rice *tab1* produces no tillers, while it normally develops the primary shoot (Tanaka *et al.* 2015). Morphological observation revealed that, in *tab1*, axillary meristem formation terminates at various points in developmental stages 1–2, resulting in the loss of axillary buds. A subsequent study found that *TAB1* promotes stem cell fate in the premeristem zone during axillary meristem formation (Fig. 4A, 4B) (Tanaka and Hirano 2020a). This is consistent with the expression pattern of *TAB1*, which is transiently observed in the central part of the premeristem zone in stages 1–2 but disappears in later stages (Fig. 4A) (Tanaka *et al.* 2015). In turn, a close paralog of *TAB1*, *OsWOX4* starts to be expressed in the established axillary meristem in the later stage (Fig. 4A, stage 3) and maintains stem cells in the meristem (Fig. 4B). As described above, in *tab1*, developmental arrest of the premeristem zone occurs during axillary meristem formation, but its formation normally initiates. Consistent with this, the initial regulators of axillary bud formation, *MOC1* and *LAX1*, are normally expressed in the *tab1* mutant (Tanaka *et al.* 2015). These observations suggest that *TAB1* is indispensable for the maintenance of stem cells, but not for their initiation. Additionally, it seems likely that *TAB1* acts downstream of *MOC1* and *LAX1*, or in an independent pathway.

In contrast to *TAB1*, *FON2* negatively regulates stem cell fate during axillary bud formation (Fig. 4B) (Tanaka and Hirano 2020a, 2020b). In the *fon2* mutant, regions, where its own transcript (used as a stem cell marker) and the *TAB1* transcript accumulate in the premeristem zone, are expanded, whereas, in the *FON2* overexpressor, axillary meristem formation is inhibited due to failed maintenance of undifferentiated cells. This suggests that *FON2* restricts the stem cell population at the appropriate size by moderately repressing *TAB1* expression to properly proceed with axillary meristem formation (Fig. 4B). Interestingly, the *tab1 fon2* double mutant produces a few tillers, indicating that the *fon2* mutation partially rescues the *tab1* phenotype (Tanaka and Hirano 2020a). This is in contrast to the genetic relationship between *WUS* and *CLV3* in *Arabidopsis*, in which the *wus* mutation is epistatic to the *clv3* mutation (Schoof *et al.* 2000). The interesting phenotype of *tab1 fon2* is explained by the precocious function of *OsWOX4*: in the absence of both *TAB1* and *FON2* function, *OsWOX4* is expressed precociously during axillary bud formation (Tanaka and Hirano 2020a). In fact, when *OsWOX4* was artificially expressed in the early stage during axillary bud formation by using the *TAB1* promoter in *tab1* mutant background, tiller formation was usually observed, implying that *OsWOX4* probably substitutes for the *TAB1* function. Therefore, *OsWOX4* is likely to be negatively regulated by the cooperative action of *TAB1* and *FON2* in the early stage (Fig. 4B). It will be interesting to understand the molecular basis of the switch in function from *TAB1* to *OsWOX4* during axillary bud formation.

### Regulation of tiller outgrowth

Axillary bud elongation determines the number of tillers which will be effective to produce panicles and hence, is an important aspect regulating rice plant architecture. Axillary bud elongation has been extensively studied in the context of apical dominance, a phenomenon in which the primary shoot represses the growth of axillary buds. Studies in various species demonstrated that auxin is a major hormone involved in apical dominance (Barbier *et al.* 2019, Domagalska and Leyser 2011, Muller and Leyser 2011, Wang *et al.* 2018a). Auxin regulates the accumulation of two other hormones cytokinin and strigolactone which activate and repress the growth of axillary buds, respectively (Domagalska and Leyser 2011). Sugar and nitrogen supply also regulate this process (Barbier *et al.* 2019, Wang *et al.* 2018a). Importantly, these hormones and nutrients often cross-regulate each other and converge on their downstream transcription factors. Thus, tiller outgrowth is a process regulated by the crosstalk among multiple hormones and nutrients.

#### Hormonal control of tiller outgrowth

Auxin is synthesized in young leaves and polarly transported down the stem by the cooperative actions of auxin influx and efflux carriers (Domagalska and Leyser 2011). Based on the studies in *Arabidopsis*, auxin efflux carrier AtPIN1 is specifically expressed in xylem parenchyma and cambium cells of the stem and forms a major stream of polar auxin transport (PAT) downward from shoot apices to stems (Domagalska and Leyser 2011). On the other hand, other PIN proteins AtPIN3, AtPIN4 and AtPIN7, which are expressed in broader regions of the stem, constitute less polar and more diffusive auxin flow called “connective auxin transport (CAT)” (van Rongen *et al.* 2019). A strong PAT in the stem vasculatures is considered to inhibit the formation of a new sink of auxin in surrounding regions, which is required for vascular connection from younger axillary buds. Once the PAT is weakened by decapitation (removal of the primary shoot), self-reinforcing canalization of auxin flow is considered to establish new paths between axillary buds and the stem vasculatures through CAT (van Rongen *et al.* 2019). In rice, several studies have indicated the involvement of PIN efflux carriers in tiller outgrowth. Knockdown and knockout mutants of AtPIN1 orthologues in rice, *OsPIN1a* and *OsPIN1b*, increase tiller numbers (Li *et al.* 2019, Xu *et al.* 2005). On the other hand, overexpression of *OsPIN2* increased tiller outgrowth (Chen *et al.* 2012). In addition, overexpression and knockout of *OsPIN9* result in increased and decreased tillering, respectively (Hou *et al.* 2021). Interestingly, *OsPIN9* expression is induced by ammonium, a major source of nitrogen in paddy fields, suggesting a regulatory link between auxin transport and nitrogen supply (for the effect of nitrogen on tillering, see subsection “*Effects of nitrogen and sugar on tiller outgrowth*”) (Hou *et al.* 2021). Although the detailed expression patterns of these PIN proteins remain to be studied, these results suggest that PIN proteins in rice are also functionally differentiated in the context of the regulation of tiller outgrowth.

CK is considered to promote bud elongation (Fig. 4C) (Muller *et al.* 2015, Sachs and Thimann 1967). Although this hormone is known to be synthesized in roots and transported to the shoot-ward direction, it is also produced locally in shoots (Nordstrom *et al.* 2004). Auxin downregulates the expression of *IPT* family genes to repress CK biosynthesis in shoots (Minakuchi *et al.* 2010, Tanaka *et al.* 2006). Upon auxin depletion in the stem, CK may be locally produced and stimulate bud elongation as a second messenger of depleted auxin. In addition, multiple members of cytokinin oxidase genes including *OsCKX4*, *OsCKX9* and *OsCKX11* are reported to repress tiller outgrowth through CK degradation in rice (Duan *et al.* 2019, Wang *et al.* 2022a, Zhang *et al.* 2021). These studies suggest that CK acts as a positive regulator of tiller outgrowth, although its downstream events in tiller outgrowth are largely elusive.

Strigolactone (SL) also acts as a second messenger of auxin, but in this case, to inhibit tiller outgrowth (Fig. 4C). SL is synthesized mainly in the roots, transported acropetally from the roots into the shoots, and moves into axillary buds to inhibit its elongation (Gomez-Roldan *et al.* 2008, Umehara *et al.* 2008). Mutants in SL biosynthesis and signaling pathways commonly show so-called “tillering dwarf” phenotypes, in which repression of tiller outgrowth is compromised. SL production is promoted by auxin through the activation of a biosynthetic enzyme gene *DWARF10* (*D10*), encoding a CAROTENOID CLEAVAGE DIOXYGENASE 8 (CCD8), in vasculatures of various organs (Arite *et al.* 2007). SL perception causes the degradation of a downstream repressor protein DWARF53 (D53) in rice. D53 is a nuclear protein which recruits TOPLESS-related transcriptional co-repressors to repress downstream genes (Jiang *et al.* 2013). D53 physically interacts with IDEAL PLANT ARCHITECTURE1 (IPA1)/WEALTHY FARMER’S PANICLE (WFP) to inhibit its transcriptional activity (Fig. 4C). *IPA1/WFP* was identified as a key regulator of tiller numbers and panicle branching and encodes an SPL family transcription factor (Jiao *et al.* 2010, Miura *et al.* 2010). Loss of function mutants of *IPA1* show the high tillering and dwarf phenotype and are insensitive to the exogenous application of SL (Song *et al.* 2017). IPA1 interacts with D53 and binds to the promoter of D53 in absence of SL. Upon SL treatment and subsequent D53 protein degradation, IPA1 activity is de-repressed and transcription of *D53* is activated, forming a feedback loop of SL signaling. A recent study revealed OsBZR1, a transcription factor downstream of brassinosteroid (BR) signaling, recruits D53 to repress the expression of a tillering repressor, *OsTEOSINTE BRANCHED1* (*OsTB1*)/*FINE CULM1* (*FC1*) and activates tiller growth (Fig. 4C) (about *OsTB1*/*FC1*, see the next subsection). BR is known to stabilize the OsBZR1 protein, whereas SL treatment triggers the degradation of not only D53 but also OsBZR1 (Fang *et al.* 2020). Importantly, BR-deficient or insensitive mutants commonly show a reduced tillering, and these mutations mask the high tillering phenotype of SL-related mutants, indicating an essential role of the BR pathway in promoting tiller growth downstream of SL signaling. In addition, the above-mentioned *OsCKX9* is also a likely target of the SL pathway. Unique to *OsCKX9* is that its expression is triggered by SL, whereas most of the other *OsCKX*s are activated by CK (Duan *et al.* 2019). Taking together, SL plays a central role to repress tiller outgrowth through the crosstalk among multiple hormones and key transcription factors.

GA is an additional phytohormone known to negatively regulate tiller outgrowth. GA-deficient and GA-signaling mutants produce more tillers than the wild type, while a loss-of-function mutant of the GA-signaling repressor, *SLENDER RICE1* (*SLR1*), and an overexpressor of the GA biosynthesis gene exhibit fewer tillers (Ikeda *et al.* 2001, Liao *et al.* 2019). Histological analysis found that these tiller phenotypes are dependent on the extent of the bud outgrowth, suggesting that GA negatively controls axillary bud elongation (Liao *et al.* 2019). GA is perceived by its receptor GIBBERELLIN INSENSITIVE DWARF1 (GID1), leading to the destruction of SLR1 through the F-box protein GID2-mediated 26S proteasome pathway (Hirano *et al.* 2008, Sasaki *et al.* 2003, Ueguchi-Tanaka *et al.* 2005). Recent studies revealed a mechanistic link between GA and *MOC1*, a crucial regulator of axillary bud formation. Overexpression of *MOC1* leads to the formation of higher-order tillers such as tertiary, quaternary, and quinary ones, that are rarely observed in wild type, suggesting that *MOC1* promotes not only bud formation but also its outgrowth (Li *et al.* 2003). The stability of the MOC1 protein is regulated by TILLERING AND DWARF1 (TAD1), a co-activator of the anaphase-promoting complex (APC/C), a multi-subunit E3 ligase. A loss-of-function mutant of *TAD1* shows an increased tiller phenotype with an increased protein level of MOC1, while *TAD1* overexpressors display a reduced tiller number with a reduced MOC1 accumulation (Xu *et al.* 2012). This relationship is explained by the regulatory mechanism in which TAD1 recruits MOC1 to the APC/C by interacting with OsAPC10 and then degrades MOC1 in a cell cycle-dependent manner (Fig. 4C). SLR1, a GA-signaling repressor, was recently shown to interact with MOC1 to prevent its degradation independent of TAD1. The protein level of SLR1 correlates with that of MOC1 and both proteins are degraded upon GA treatments, providing an explanation for the inhibition of tiller outgrowth by GA (Fig. 4C) (Liao *et al.* 2019). Besides, the GA pathway also restricts tillering by interfering with the effect of nitrogen, an essential component of soil nutrients for plant growth. We will deal with this point in subsection “*Effects of nitrogen and sugar on tiller outgrowth*”.

#### Function of *OsTEOSINTE BRANCHED1 (FINE CULM1)* as a central hub of bud elongation

*teosinte branched1* (*tb1*) is responsible for one of the classical QTLs that determine the difference in plant architecture between modern maize, which develops a few tillers, and its highly tillered ancestor, teosinte (Doebley *et al.* 1990, 1995, Doebley and Stec 1991, 1993). Regulatory elements selected during maize domestication led to increased expression of *tb1*, resulting in strong apical dominance (Doebley *et al.* 1997, Wang *et al.* 1999). *tb1* belongs to the *TCP* (*TEOSINTE BRANCHED1*, *CYCLOIDEA*, *PCF*) gene family, whose members encode plant-specific transcription factors. Genetic studies revealed that the *tb1* function inhibiting bud elongation is widely conserved among angiosperms. For example in *Arabidopsis*, two orthologs of *tb1*, *BRANCHED1* (*BRC1*) and *BRC2* act redundantly to repress bud outgrowth (Aguilar-Martínez *et al.* 2007).

In rice, *OsTB1*, which is also referred to as *FINE CULM1* (*FC1*), was identified based on its sequence similarity to the maize *TB1* (Takeda *et al.* 2003). The overexpressor of *OsTB1* showed a significant reduction in tiller outgrowth, whereas a loss-of-function mutant of *OsTB1*, known as *fc1*, causes an increase in the number of tillers (Takeda *et al.* 2003). These phenotypes indicate that *OsTB1* negatively regulates axillary bud elongation during tiller formation (Fig. 4C). Consistent with this function, *OsTB1* is expressed in the axillary bud including the meristem and leaf primordia (Minakuchi *et al.* 2010, Takeda *et al.* 2003). Further genetic and physiological analyses have revealed the relationship between the actions of *OsTB1* and SL. The exogenous application of SL to the wild-type plant inhibited bud elongation, while its application to the *fc1* mutant did not affect its tiller phenotype (Minakuchi *et al.* 2010). In addition, overexpression of *OsTB1* partially suppressed the tiller-overproducing phenotype of *dwarf3* (*d3*), a mutant of the gene encoding an F-box protein involved in the SL signaling pathway. These observations suggest that *OsTB1* partially acts downstream of SL to regulate bud elongation.

Recently, much information about *tb1*/*BRC1*, *2*/*OsTB1* has accumulated and their essential roles as integrators of multiple signaling pathways came into view (Fig. 4C) (Wang *et al.* 2019). Auxin-induced apical dominance requires the activity of *BRC1* (Aguilar-Martínez *et al.* 2007), while CK is likely to be involved in tiller formation partly via negative regulation of *OsTB1* (Minakuchi *et al.* 2010). As mentioned above, the perception of SL by their receptor leads to the degradation of the D53-OsBZR1 complex to promote *OsTB1* transcription (Fang *et al.* 2020). IPA1/WFP negatively regulates bud elongation by promoting the expression of *OsTB1* by directly binding to its promoter (Lu *et al.* 2013). Nitrogen fertilizer causes the downregulation of *OsTB1* to promote tiller formation (Li *et al.* 2016). In addition, OsTB1 positively regulate the expression of *DWARF14* (*D14*), which encodes an SL receptor (Arite *et al.* 2009, Guo *et al.* 2013, Nakamura *et al.* 2013). All these studies highlight the importance of *tb1*/*BRC1*, *2*/*OsTB1* (*FC1*) as a central hub to control bud elongation in response to both plant-endogenous and exogenous cues.

A recent study reported that a paralog of *OsTB1*, *OsTB2* (also referred to as *RETARDED PALEA1*), has obtained an antagonistic function to *OsTB1* (Fig. 4C) (Lyu *et al.* 2020). Association analyses between genotypes and tiller numbers among indica and japonica cultivars including upland rice identified *OsTB2* as a regulator which promotes tillering in japonica. The japonica allele of *OsTB2* possesses a 3 bp insertion, resulting in one amino acid insertion near the TCP domain. This *OsTB2^3bp+^* allele reduces the expression of *D14* and promotes axillary bud elongation (Lyu *et al.* 2020). OsTB2 protein directly interacts with OsTB1 in planta and possibly interferes with the positive regulatory effect of OsTB1 on *D14*. This study also revealed another SNP in the 3ʹ-UTR of *OsTB2* which is likely to have evolved from the *OsTB2^3bp+^* allele in japonica cultivars. This SNP was frequently found in upland rice cultivars and associated with the reduced expression of *OsTB2*. It has been pointed out that upland rice tends to have fewer but larger tillers with better-developed root systems, possibly to adapt to dryland conditions (Fukai and Cooper 1995, Kato and Katsura 2014). Thus, the SNP that reduced *OsTB2* expression might have been utilized in upland rice to limit tiller numbers (Lyu *et al.* 2020).

#### Effects of nitrogen and sugar on tiller outgrowth

Nitrogen is an essential component of soil nutrients for plant growth and promotes tillering. In the rice Green Revolution, a null allele of *SEMIDWARF1* (*SD1*), a gene encoding a GA biosynthesis enzyme gibberellin 20-oxidase 2 (OsGA20ox2), was selected to avoid lodging, the bending over of the stem near the ground (Ferrero-Serrano *et al.* 2019, Liu *et al.* 2018, Monna *et al.* 2002, Sasaki *et al.* 2002). Besides its famous semidwarf phenotype, varieties containing the *sd1* allele are known to produce more tillers (Wu *et al.* 2020). However, the increased ability of tillering associated with the *sd1* mutation resulted in the extensive application of nitrogen fertilizers to maximize yield, causing environmental problems such as soil acidification and water eutrophication (Guo *et al.* 2010, Zhang *et al.* 2015). Therefore, the elucidation of mechanisms underlying nitrogen-stimulated tillering is of great importance for agriculture in practice.

Besides the above-mentioned *OsPIN9* activation and *OsTB1* repression (Hou *et al.* 2021, Li *et al.* 2016), recent studies identified two important regulators of tiller outgrowth in response to nitrogen (Fig. 4C). APETALA2 (AP2) domain transcription factor NITROGEN-MEDIATED TILLER GROWTH RESPONSE 5 (NGR5) promotes tillering in response to nitrogen supply by directly repressing the expression of SL-signaling genes and tillering repressors such as *IPA1* and *OsTB1* (Wu *et al.* 2020). The *ngr5* loss-of-function mutant failed to induce tiller outgrowth in response to nitrogen, whereas the allele with higher *NGR5* expression increased the number of tillers and yield (Wu *et al.* 2020). Importantly, NGR5 interacts with multiple GA signaling components including the signaling repressor SLR1, the GA receptor GID1 and the F-box protein GID2 and is destructed upon GA treatments through the proteasome pathway (Wu *et al.* 2020). In this context, SLR1 competes with NGR5 for GID1 and prevents NGR5 from GA-mediated proteolysis. Thus, this mechanism, with the above-mentioned MOC1-SLR1 module, provides explanations of how GA inhibits tillering (Liao *et al.* 2019, Wu *et al.* 2020) (Fig. 4C). Another study identified *OsTCP19* as a tillering repressor in response to nitrogen supply (Liu *et al.* 2021). The *OsTCP19* expression is induced at low nitrogen levels but is repressed at higher concentrations (Fig. 4C). OsTCP19 directly represses the expression of *DWARF AND LOW TILLERING* (*DLT*), an important BR-signaling gene to promote tiller outgrowth (Liu *et al.* 2021, Tong *et al.* 2009). Many accessions including modern japonica and indica cultivars collected from the areas/countries with high soil nitrogen levels possessed a 29-bp insertion in the *OsTCP19* promoter. This insertion attenuates the repression of *OsTCP19* expression by nitrogen and therefore confers a reduced tillering response to the nitrogen supply. In contrast, most accessions from areas with low soil nitrogen levels and most *O. rufipogon* accessions do not have this insertion and show high tillering in response to nitrogen (Liu *et al.* 2021). Importantly, the introgression of this *OsTCP19* allele with the high tillering response into modern cultivars successfully increased grain yield at low levels of nitrogen supply (Liu *et al.* 2021). Studies mentioned here provide promising strategies to improve nitrogen use efficiency which can contribute to sustainable agriculture with a reduced environmental burden.

Sugars are also known as key regulators of axillary bud elongation (Barbier *et al.* 2019). For a long time, auxin was recognized as a primary regulator of apical dominance. However, a study using *Pisum sativum* (pea) has revealed that the demand for sucrose, but not auxin, in the primary shoot tip restricts sugar availability to axillary buds and their growth (Mason *et al.* 2014). This study also found that sucrose partially repressed the expression of *PsBRC1*, an ortholog of *tb1*/*BRC1*, *2*/*OsTB1* to promote bud elongation. Rice *MONOCULM2* (*MOC2*) encodes a fructose-1,6-bisphosphatase1 that is responsible for the sucrose biosynthesis pathway (Koumoto *et al.* 2013). Loss of *MOC2* causes a significant reduction in tiller number, probably due to a shortage of sucrose supply to axillary buds, supporting the importance of sugars in controlling bud elongation also in rice (Fig. 4C).

### Control of tiller angle

The tiller angle is one of the most important factors determining the shoot architecture. It is controlled by the extent of growth on the upper and lower sides of the tiller base. Symmetric growth on both sides results in a prostrate habit with larger tiller angles which is often observed in wild rice species. This growth habit is advantageous for occupying a larger ground area and possibly for avoiding infestation of pathogens and insects (Wang *et al.* 2022b). Asymmetric growth with a greater extent in the lower side of the tiller base, on the other hand, causes erect habits with smaller tiller angles. This trait is preferred in agriculture because it increases planting density per unit area. Regulation of tiller angles involves multiple intrinsic and extrinsic factors such as gravitropism, phytohormones, growth phase, and temperature (Wang *et al.* 2022b). Here, we briefly introduce our current understanding of the core components of the gravitropism pathway and how its modification affects tiller angles. We also deal with major transitions from the prostrate to erect growth during domestication and post-domestication.

#### Components for shoot gravitropism

Shoots and roots typically grow upward and downward, respectively. These growth directions are defined by gravity and the process of this growth control is called gravitropism. As gravitropism plays pivotal roles in the regulation of growth angles both in tillers and roots, we first briefly describe the central components of this pathway.

Gravity-sensing is considered to occur in specific cell types in which starch-filled amyloplasts sediment toward the gravity vector. These gravity-sensing cells can be found in several rice tissues including coleoptile, leaf sheath bases and columella root caps (Wang *et al.* 2022b). The sedimentation of amyloplasts is considered to be the initial step of gravity sensing both in shoots and roots. Accumulation of starch granules is important to facilitate sedimentation of amyloplasts and hence gravitropism, however, it is not essential for gravity sensing because starch-deficient mutants still show a residual response to gravity (Huang *et al.* 2021b, Okamura *et al.* 2013, 2014). The sedimentation of amyloplasts is sensed by yet unknown mechanisms and causes the accumulation of auxin at the lower side of organs (Wang *et al.* 2022b). In *Arabidopsis*, this asymmetric distribution of auxin is mediated by a shift of PIN3 localization to the bottom side of the membrane in gravity-sensing cells (Friml *et al.* 2002). Subsequently, a high level of auxin at the bottom side of organs triggers differential growth between upper and lower sides, resulting in upward and downward growth in shoots and roots, respectively (Jiao *et al.* 2020).

LAZY1 is a central component in this auxin re-distribution and was discovered from the study of a recessive rice mutant *lazy1* (Li *et al.* 2007, Yoshihara and Iino 2007). *lazy1* mutants show larger tiller angles due to a failure to form the asymmetrical auxin distribution during gravitropism response in shoots. *LAZY1* encodes a protein with five conserved domains and belongs to one of two large clades of the IGT (named after a conserved motif) protein family (Dardick *et al.* 2013). There are two subclades among LAZY1 homologs; genes in one subclade including *LAZY1* are preferentially expressed in shoots, whereas those in another subclade are important for gravitropism not only in shoots but also in roots (Jiao *et al.* 2020) (See the section “Root”). A recent study in *Arabidopsis* roots showed that AtLZY3 polarly localizes on the bottom side of the columella cell membrane shortly after gravity stimuli, and recruits RCC-like domain proteins (RLDs) (Furutani *et al.* 2020). RLDs are novel regulators of auxin transport and are required for PIN3 accumulation toward the lower side of rotated roots (Furutani *et al.* 2020). This study provides an explanation for the function of LAZY proteins in creating an asymmetric auxin distribution for gravitropism.

There is another clade of IGT family proteins lacking the C-terminal conserved domain. Rice *TILLER ANGLE CONTROL1* (*TAC1*) is the first gene identified which belongs to this clade (Yu *et al.* 2007). Japonica cultivars generally show narrower tiller angles compared to indica cultivars. A loss-of-function allele of *TAC1* is widely shared in japonica cultivars, whereas the majority of indica cultivars with relaxed tiller angles have its functional allele, indicating that *TAC1* increases tiller angles. Similarly, in *Prunus persica* (peach) and *Arabidopsis*, mutations of *TAC1* orthologs cause narrower branch angles (Dardick *et al.* 2013). The *TAC1* clade genes exist only in vascular plants with axillary branches, whereas *LAZY1* genes are found commonly in terrestrial plant lineages. This suggests that *TAC1* genes had evolved from the *LAZY1* clade through the truncation of the C-terminal domain to balance the effect of LAZY1 proteins in the regulation of branch angles (Dardick *et al.* 2013).

#### Modification of tiller angles during domestication and post-domestication

Rice cultivars commonly have small tiller angles, whereas their ancestor *O. rufipogon* shows a diverse variation in growth habits from erect to prostrate (Jin *et al.* 2008, Tan *et al.* 2008). This indicates that the erect growth habit was selected during rice domestication. *PROSTRATE GROWTH 1* (*PROG1*) is the first regulator of tiller angles identified from *O. rufipogon* (Jin *et al.* 2008, Tan *et al.* 2008). It encodes a C2H2 zinc finger transcription factor which increases the tiller angle by promoting equal growth on both the upper and lower side of the tiller base. Later, it turned out that there is a cluster of similar genes named *RICE PLANT ARCHITECTURE DOMESTICATION* (*RPAD*) in the vicinity of *PROG1* in wild species, but a large (~110 kb) deletion eliminating the cluster had occurred in cultivars (Wu *et al.* 2018). Some genes in this cluster have functions similar to *PROG1*, suggesting that prostrate growth is controlled by multiple redundant genes in wild species. Importantly, all cultivars examined so far have an identical allele of loss-of-function of *PROG1* and the *RPAD* deletion, suggesting this allele had undergone a strong artificial selection to eliminate prostrate growth during domestication (Wu *et al.* 2018).

Interestingly, a highly similar elimination of this locus occurred in African domesticated rice *O. glaberrima*, which was domesticated from *O. barthii* (Hu *et al.* 2018, Wu *et al.* 2018). Therefore, these studies are examples of parallel domestication and indicate that the selection of erect growth habits was an indispensable step during rice domestication. Besides the function of controlling the tiller angle, these genes have pleiotropic roles in increasing tiller number and decreasing panicle branching, suggesting that the loss of this gene cluster contributed in multiple ways to modifying rice plant architecture (Tan *et al.* 2008).

Although the loss of *PROG1* and *RPAD* loci is highly shared among cultivars, the identification of additional regulators for tiller angles revealed distinct routes of genetic modifications between japonica and indica for this trait. Indica varieties usually show relaxed tiller angles compared to japonica varieties (Yu *et al.* 2007). QTL analyses focused on these variations yielded the identification of *TAC1* as mentioned above. *TAC3* and *D2*, encoding a plant-specific protein with unknown function and a cytochrome P450 enzyme involved in the BR synthesis, respectively, were identified as two additional QTLs explaining the smaller tiller angles in japonica (Dong *et al.* 2016). Although causal polymorphisms for these two QTLs have not been determined yet, this study suggested that alleles with reduced functions were selected in japonica. On the other hand, *TILLER INCLINED GROWTH 1* (*TIG1*), encoding a TCP transcription factor closely related to *OsTB1*, was identified from a cross between indica cultivar 93-11 and *O. rufipogon* (Zhang *et al.* 2019). In 93-11, the expression of *TIG1* was lost due to mutations in its promoter region. This recessive *tig1* allele was widespread among indica accessions, whereas the functional *TIG1* allele is common in *O. rufipogon* and japonica varieties. Thus, tiller angles had been modified in two distinct ways; loss of function mutations of *TAC1*, *TAC3* and *D2* in japonica and that of *TIG1* in indica were selected independently after the initial step of domestication associated with loss of *PROG1* and *RPAD*.

## Stem

The stem is the plant shoot axis and consists of nodes and internodes alternately. Nodes are attachment points of lateral organs such as leaves and flowers to the axis in which complex vascular networks are established between these organs. Internodes are elongating portions formed between nodes and lift leaves for photosynthesis (Hoshikawa 1989, Takane and Hoshikawa 1993). Due to the agronomic importance of lodging resistance, regulatory mechanisms of internode elongation are well studied as exemplified by the GA pathway utilized in Green Revolution (Ferrero-Serrano *et al.* 2019, Liu *et al.* 2018, Monna *et al.* 2002, Sasaki *et al.* 2002). On the other hand, although the stem is one of the major organs commonly seen in seed plants, its developmental process has been largely elusive. In this section, we describe the rice stem structure briefly and discuss our current understanding of the regulation of stem development. We also highlight recent studies using deepwater rice that revealed the unique strategy involving stem growth in adaptation.

### The stem structure in rice

**Fig. 5.**
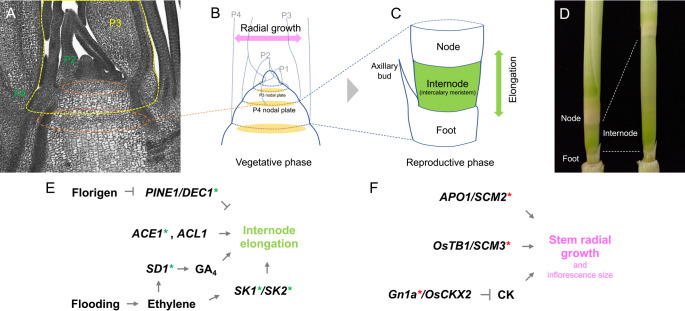
Developmental processes of the stem and their regulators. (A) A tissue section at the vegetative shoot apex. A dotted line in yellow represents a putative unit of the stem and P3 leaf primordium. Orange dotted circles are nodal plates. (B) A schematic representation of the vegetative stems shown in (A). (C) A schematic representation of the elongating stem in the reproductive phase. (D) A photograph illustrating that internodes are the primary part contributing to stem elongation. (E and F) Regulatory pathways for the internode elongation (E) and the radial growth of the stem (F). Green asterisks represent genes/QTLs found in deepwater rice. Red asterisks are QTLs that promote stem radial growth.

A portion of the stem between two successive leaves is a building unit along the plant axis. In this unit, the rice stem can be further divided into three parts; the upper and lower growth-limited parts and an elongating one in between them (Fig. 5C, 5D). The elongating part is an internode that has a large central lacuna and accumulates chlorophyll. The upper non-elongating part is a node where a leaf attaches and complex vascular networks develop inside (Hoshikawa 1989). The lower part beneath the internode is unnamed, therefore, we tentatively call this region a “foot” (Fig. 5C). The foot shares the central lacuna continuous from the internode at maturity, however, it is also similar to the node because it does not significantly elongate, is chlorophyll-less and develops complex vascular networks between the stem and axillary buds (Fig. 5D) (Takane and Hoshikawa 1993). Thus, the foot may be a part of the internode but it is appropriate to treat the foot separately from the internode because it expresses multiple distinct characters.

Histologically, we can recognize the node and internode pattern in the center from the P3 stem onward (Fig. 5A) (Kaufman 1959). The nodal region is called a “nodal plate”, which consists of isotropic and smaller cells compared to those in the internode (Fig. 5A, 5B). In internodes, anticlinal cell division is apparent, forming vertical files of vacuolated cells (Fig. 5A). Meanwhile, the radial and longitudinal growth occur in the periphery of the stem through periclinal and anticlinal cell divisions in the ground meristem. At this stage, there is no clear distinction in this area along the longitudinal axis (Kaufman 1959). At later stages, a zone of extensive anticlinal cell division, so-called the “intercalary meristem”, is formed in the middle region along the longitudinal axis (Fig. 5C). The distinctions between the node, internode and foot are unclear until then from the outer appearance of the stem.

In typical rice cultivars, internodes are not evident during the early vegetative phase and hence the boundary between the node and the foot is unclear (Hoshikawa 1989, Takane and Hoshikawa 1993). For the same reason, elongation of the stem is quite limited at young stages. Upon reproductive transition, the stem starts developing internodes and its elongation becomes evident in the last four or five phytomers.

### Coordination of internode elongation with reproductive growth

A recent study revealed an important regulator of this transition (Gomez-Ariza *et al.* 2019). Knockout mutants of a C2H2 zinc finger transcription factor gene named *PREMATURE INTERNODE ELONGATION1* (*PINE1*) showed ectopic internode elongation in early vegetative shoots. The mutant internodes can elongate further in response to GA treatment, whereas wild-type stems are not at this stage. *PINE1* is highly expressed in vegetative stems but is down-regulated by rice florigen genes *Heading date 3a* (*Hd3a*) and *RICE FLOWERING LOCUS T1* (*RFT1*) (Fig. 5E). Thus, *PINE1* negatively regulates GA response and its down-regulation is important to coordinate stem elongation with inflorescence development in the reproductive phase.

### Regulation of internode elongation in deepwater rice

In Southeast Asia, severe flooding occurs during the monsoon season. Deepwater rice is a subsistence crop in such flood-prone areas and possesses the ability to rapidly elongate its internodes to keep leaves above the water level to avoid drowning (Kende *et al.* 1998). A pioneering work in the genetics of flood-induced internode elongation in deepwater rice is the identification of *SNOKEL1* (*SK1*) and *SK2* (Hattori *et al.* 2009). QTL analysis between a deepwater cultivar C9285 and non-deepwater cultivar Taichung 65 (T65) detected three QTLs on chromosomes 1, 3 and 12. The detailed mapping of the QTL with the largest effect on the chr.12 revealed that C9285 has two AP2/ERF domain containing transcription factors SK1 and SK2. Both genes are activated by the accumulation of the gaseous hormone ethylene in response to flooding. SK1 and SK2, in turn, activate GA production for internode elongation (Hattori *et al.* 2009). DNA sequence comparison among wild rice accessions suggested that functional *SK2* is present not only in *O. rufipogon*, but also in *O. glumaepatula*, a species in the Amazon River area in South America where seasonal flooding also occurs (Hattori *et al.* 2009). This suggests that *SK2* is especially important for internode elongation in response to flooding.

A subsequent study of the QTL on chr.1 identified *SD1* as a causal gene (Kuroha *et al.* 2018). The GA20 oxidase2 encoded by *SD1* catalyzes the limiting step of GA biosynthesis and its mutation which greatly reduced its catalytic activity results in shorter internodes and higher lodging resistance (Monna *et al.* 2002, Sasaki *et al.* 2002). Interestingly, *SD1* was utilized in the opposite way in deepwater rice. Flooding-induced elevation of the ethylene accumulation activates OsEIL1a, a transcription factor involved in ethylene signaling. It then directly activates *SD1*. Although the OsEIL1a binding site is identical to that of normal cultivars, the *SD1* allele in deepwater rice is capable of increasing its transcription in response to ethylene signaling (Fig. 5E) (Kuroha *et al.* 2018). It is worth noting that the GA20 oxidase from this allele promotes the accumulation of GA_4_ rather than GA_1_ and that GA_4_ has a higher activity to elongate internode than GA_1_. This study also revealed the possible origin of this *SD1* allele in *O. rufipogon* accessions from Bangladesh (Kuroha *et al.* 2018).

A further study by the same group identified the causal genes for two additional QTLs. A new QTL on chr.12, physically linked to *SK1* and *SK2*, was named *DECELERATOR OF INTERNODE ELONGATION1* (*DEC1*) (Nagai *et al.* 2020). Interestingly, the *DEC1* is the same gene as *PINE1* and the allele in deepwater rice is repressed by GA or flooding treatment, whereas that in normal cultivars is insensitive and constantly active in the young shoot. There is a large deletion in the *PINE1*/*DEC1* promoter which might have resulted in constitutively active expression in normal cultivars (Nagai *et al.* 2020). Deepwater rice has another regulator *ACCELERATOR OF INTERNODE ELONGATION1* (*ACE1*) on chr.3, which encodes a nuclear protein with an unknown function. *ACE1* confers responsiveness to GA exogenically applied or increased by flooding treatment to promote internode elongation in deepwater rice (Fig. 5E) (Nagai *et al.* 2020). In normal cultivars, *ACE1* is nonfunctional but genes homologous to *ACE1* are likely to play redundant roles in internode elongation during the reproductive transition. These studies suggested that the regulation of GA response through *PINE1*/*DEC1*, *ACE1* and its homologs is a key step to trigger internode initiation and/or elongation not only in deepwater rice but also in normal cultivars.

### QTLs determining culm diameter

Radial growth of the stem determines the diameter of the culms (elongating internodes) which is an important trait for lodging resistance. Ookawa and colleagues reported *STRONG CULM2* (*SCM2*), a major QTL for the increased stem diameter (Ookawa *et al.* 2010) (Fig. 5F). *SCM2* is a weak gain-of-function allele of *ABERRANT PANICLE* *ORGANIZATION1* (*APO1*), which encodes an F-box protein (*APO1* function in panicles is also discussed in the section “Inflorescence”). This allele shows a moderate increase in *APO1* transcript level and causes no detrimental effect on yield. The same group also identified another QTL *SCM3*, encoding *OsTB1* which is known to promote culm radial growth and repress tillering through activation of SL signaling (Guo *et al.* 2013, Minakuchi *et al.* 2010, Takeda *et al.* 2003, Yano *et al.* 2015) (Fig. 5F). *SCM3* is also a gain-of-function allele with a mild increase in *OsTB1* expression. Importantly, plants with these two QTLs show an increase in inflorescence meristem size and produce larger panicles with increased grain yield. Another recent study reported that a loss-of-function of CK inactivation enzyme gene *OsCKX2/Gn1a* also significantly increases the culm diameter and panicle size (Tu *et al.* 2022). Thus, these studies suggest that *APO1*, *OsTB1*-SL signaling, and CK are positive regulators of the radial growth at the shoot apex important not only for lodging resistance, but also for inflorescence size (see the section “Inflorescence”) (Fig. 5F). In addition, these studies also propose a gene pyramiding strategy in which QTLs with weak or mild effects in distinct molecular pathways can be successfully combined without detrimental effects on yield (Tu *et al.* 2022, Yano *et al.* 2015).

### Potentially important regulators for the stem development

Due to the easily visible phenotype, many dwarf mutants have been isolated in rice. There are many possible molecular defects which lead to dwarfism, including cell/tissue differentiation, hormone pathways, cell wall deposition and other cellular metabolisms (Liu *et al.* 2018). Besides GA mentioned above, another possible important player is BR. A loss-of-function mutant of *BRD1*, encoding a key enzyme of BR synthesis, shows an extremely dwarf phenotype with disorganized stem internal structures (Hong *et al.* 2002). Interestingly, a clear distinction between the nodal plate and vertical cell files of internode beneath the shoot apex (Fig. 5A) is absent in *brd1* mutants (Hong *et al.* 2002, 2004). Given that mutants in BR biosynthesis and signaling components commonly show dwarfisms and that this hormone promotes the transverse arrangement of cortical microtubules to accelerate cell elongation (Mayumi and Shibaoka 1995, Zhang *et al.* 2014), BR is likely to play an important role in stem tissue patterning and/or organized cell elongation based on the positional information.

Indeterminacy regulators for shoot meristems are also important in stem development. Loss-of-function mutants of *OSH15* show dwarfism with abnormal epidermal cells and vascular structures (Sato *et al.* 1999). Similarly, maize *BLH12* and *BLH14*, which encode cofactors of KN1, are required for the formation of stem and internal vascular networks (Tsuda *et al.* 2017). Consistent with these defects, both *KNOX* and *BLH* genes are expressed in ground meristems and provascular bundles in young stems (Sato *et al.* 1999, Tsuda *et al.* 2017). These studies suggest that *KNOX* and *BLH* genes regulate cell/tissue and vascular differentiation during early stem development.

Despite the importance of plant height in crop breeding, studies of stem development are limited. This is possibly due to the lack of distinct morphological landmarks at least externally during the early development of the stem (Fig. 5A). Comprehensive observation of internal vascular networks, which reflect differences between the node, internode and foot, is also challenging. Furthermore, spatial and temporal relationships among known regulators are also elusive. Future studies with advanced tissue/cell-type specific transcriptomes and imaging techniques should overcome these obstacles and unveil molecular processes of stem development.

## Inflorescence

The inflorescence architecture is the primary determinant of grain yield. *O. rufipogon* produces fewer panicle branches (especially secondary branches) compared to *O. sativa*, suggesting the increase in branching activity had occurred during domestication (Yamaki *et al.* 2010). In addition, the post-domestication breeding program selected varieties with enhanced panicle branching to further gain yield (Wing *et al.* 2018). In this section, we summarize the functions of regulators for inflorescence development and how modifications in these players contributed to improving the inflorescence architecture.

### Rice inflorescence architecture

**Fig. 6.**
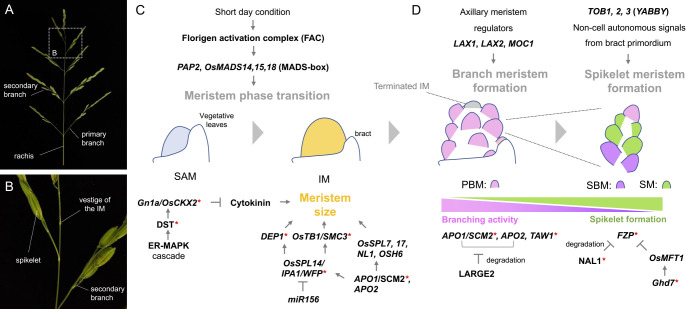
Developmental processes of rice inflorescence and their regulators. (A and B) A panicle of a japonica cultivar Taichun65. A dashed box indicates the region magnified in (B). (C) Regulatory pathways in the inflorescence meristems at the reproductive transition. (D) Regulatory pathways at the branch meristem formation stages. Red asterisks represent genes or proteins whose natural variations are known to affect inflorescence architectures.

The panicle, an inflorescence in rice, consists of the main axis called the rachis, primary and secondary branches and lateral or terminal spikelets (Fig. 6A, 6B). Upon reproductive transition, the SAM stops producing vegetative leaves and is converted to the inflorescence meristem (IM) which initiates primary branch meristems (PBM) (Fig. 6C). The IM activity is terminated after PBM formation and can be seen as a vestige at the base of the most distal primary branch at maturity (Fig. 6B, 6D) (Ikeda *et al.* 2004). In turn, PBMs produce further branch meristems while maintaining themselves at the tip. Several branch meristems at the base of primary branches will be secondary branch meristems (SBM), and those formed later in the distal region will be spikelet meristems (SM) (Fig. 6D). SBM initiates a few SMs. The ternary branch is occasionally formed in normal cultivars. PBMs and SBMs at the tip eventually turn into SMs. Each SM will generate a spikelet, a short branch bearing a flower in rice. All of these branch meristems form in the axils of bracts, which do not develop as leaf primordia and instead generate hairs covering young panicles (Ikeda *et al.* 2004). Therefore, inflorescence branching relies on the developmental programs controlling axillary meristem formation. Another key aspect to determine the extent of branching is the shift of meristem identities from those of PBM and SBM to that of SM. Taking together, inflorescence development involves mechanisms commonly used in vegetative and reproductive shoots and those activated after the reproductive transition.

### The reproductive transition of the shoot meristems

The vegetative SAM becomes the IM upon reproductive transition triggered by the florigen signals (Fig. 6C) (Tsuji 2017). *Hd3a* and *RFT1* are two florigen genes essential for flowering in rice (Komiya *et al.* 2008). *Hd3a* is responsible for flowering under short-day conditions, whereas *RFT1* expression is induced under long-day conditions (Komiya *et al.* 2008, 2009). Their protein products are transported to the young stem beneath the SAM through vascular networks and eventually enter the SAM via unknown mechanisms (Tamaki *et al.* 2007). Florigens are then perceived by 14-3-3 proteins in the cytoplasm and move into the nucleus to form the ternary florigen activation complex (FAC) with a basic leucine-zipper transcription factor OsFD1 expressed in shoot meristems (Taoka *et al.* 2011). The chemical inhibition of the protein-protein interaction between 14-3-3 and OsFD1 prevents various florigen-dependent processes *in vivo*, supporting that FAC assembly is central for florigen function (Taoka *et al.* 2022). FAC activates three *MADS-box* genes in *AP1/FUL* subfamily *OsMADS14*, *OsMADS15* and *OsMADS18* and one gene in *SEP* subfamily *PANICLE PHYTOMER2* (*PAP2*)/*OsMADS34* to convert the SAM to IM (Fig. 6C) (Kobayashi *et al.* 2010, 2012, Tamaki *et al.* 2015, Taoka *et al.* 2011). Disruption and knockdown of these *MADS-box* genes result in shoot formation instead of inflorescence (Kobayashi *et al.* 2012). In addition, *pap2* mutation partially suppresses the early flowering phenotype of *Hd3a* overexpressors (Kobayashi *et al.* 2012). These studies illustrate a molecular mechanism which invokes the reproductive program crucial both for plant life cycle and agriculture.

### Promotion of the IM growth and branching

After the reproductive transition, the IM rapidly increases its size and starts producing PBMs. Because the size of IM and duration of PBM activity have a great impact on the spikelet number per panicle, many genes involved in these developmental processes have been identified (Fig. 6C, 6D).

*ABERRANT PANICLE ORGANIZATION1* (*APO1*) encodes F-box protein similar to *Arabidopsis* UNUSUAL FLORAL ORGANS (UFO), and *APO2* is *Arabidopsis* *LEAFY* ortholog in rice. It is contrasting that these genes specify floral meristem identity in *Arabidopsis*, whereas *APO1* and *APO2* promote IM growth and inflorescence branching. Both *apo1* and *apo2* loss-of-function mutations reduce the IM size and branch numbers (Ikeda *et al.* 2005, Ikeda-Kawakatsu *et al.* 2012). In these mutants, the IM was converted into terminal spikelets after producing several PBMs. On the other hand, *apo1-D* dominant mutants or *APO1* overexpressing plants result in larger IMs and massive inflorescence branching (Ikeda-Kawakatsu *et al.* 2009). Thus, *APO1* and *APO2* promote IM growth and branching activity (Fig. 6C, 6D). The terminal spikelet phenotypes in *apo1* and *apo2* mutants suggest that the identity of the IM is programmed to shift to that of SM, however, this does not take place in the wild type because the IM aborts before the shift (Ikeda-Kawakatsu *et al.* 2009). It is possible that the temporal order of these events (IM to SM shift and IM abortion) determines the panicle branching pattern in rice and that its alteration may underlie the diversity of inflorescence architecture among grasses.

These *APO1* dominant mutants and overexpressors could not contribute yield increase due to fewer tillering and lower grain filling rates. However, *SCM2*, a natural variation of *APO1* which moderately increases its expression level (about two-fold), results in a higher yield due to an increase in secondary branch number per panicle (Ookawa *et al.* 2010). This allele was isolated as a QTL which increased the diameter of the stem (as discussed in the section “Stem”) and was also identified as a QTL that increased the primary branch length of panicles (Agata *et al.* 2020). Thus, a mild increase in *APO1* transcript level can improve stem and inflorescence traits in multiple ways. Recently, LARGE2, a HECT-domain E3 ubiquitin ligase, was identified as a post-translational regulator for APO1 and APO2 (Huang *et al.* 2021a). LARGE2 physically interacts with these proteins and reduces their stabilities (Fig. 6D). In contrast to *apo1* and *apo2* mutants, *large2* mutants show larger panicles and seeds, representing another possible factor which can be utilized in crop improvement.

A recent study by Miao and colleagues identified downstream pathways of *APO2* to regulate panicle branching and bract suppression (Miao *et al.* 2022). APO2 directly activate *OsSPL7*, *OsSPL14*, *OsSPL17*, *NECK* *LEAF1* (*NL1*) and *ORYZA SATIVA HOMEOBOX6* (*OSH6*). Knockout mutants of these target genes showed shared phenotypes including bract overgrowth and reduction in the IM size. Consistent with this, the *osspl14* mutation suppressed an enlarged inflorescence phenotype in *35S:APO2* *apo1-D* background and overexpression of *mOsSPL14*, a *miR156* resistant version of *OsSPL14*, recovers inflorescence branching in *apo2*-1 background. These results strongly suggest that *APO2* promotes IM growth and branching through the activation of a set of transcription factors, especially *OsSPL14* (Fig. 6C) (Miao *et al.* 2022).

*OsSPL14* is known as *IPA1/WFP*, a pleiotropic gene affecting tillering and panicle branching. It is negatively regulated by *miR156*. Increased accumulation of *OsSPL14* transcript due to mutation in the *miR156*-target site or epigenetic polymorphisms results in a phenotype with fewer unproductive tillers and an increased panicle branching, regarded as the ideal plant architecture (Jiao *et al.* 2010, Miura *et al.* 2010). As discussed in sections “Tiller” and “Stem”, IPA1 directly activates *OsTB1*/*FC1*/*SCM3* which in turn represses tillering and increases stem diameter as well as panicle size (Lu *et al.* 2013, Yano *et al.* 2015). Thus, the *IPA1*-*OsTB1* module explains fewer tillering and increased panicle branching phenotypes in *ipa1* gain-of-function mutants (Fig. 6C). IPA1 also directly activates *DENSE AND ERECT PANICLE1* (*DEP1*), another key regulator of inflorescence branching. *DEP1* encodes a PEBP-domain-containing protein and was found as the dominant factor causing highly branched and compact panicle architecture (Huang *et al.* 2009). The causal allele lacks its C-terminal domain and acts in a dominant negative manner. Interestingly, a similar mutation had occurred in wheat, suggesting the same gene was selected in the similar way in different cereal crops for compact inflorescences (Huang *et al.* 2009).

*TAWAWA1* (*TAW1*) is another regulator which inhibits phase transition from branch meristems to SMs (Yoshida *et al.* 2013). *TAW1* was identified in a study of semi-dominant mutants in which transposon insertions in the promoter resulted in an increased expression from these semi-dominant alleles. It encodes an Arabidopsis LSH1 and Oryza G1 (ALOG) family transcription factor highly expressed in branch meristems. *TAW1* positively regulate *SVP* family *MADS-box* genes *OsMADS22*, *OsMADS47* and *OsMADS55* (Yoshida *et al.* 2013). Overexpression of these genes also resulted in higher branching, suggesting that *TAW1* inhibits the phase change to SM through these *MADS-box* genes. Recently, a natural variation in the *TAW1* locus was also found as a QTL which increased panicle branching in an indica cultivar (Yuan *et al.* 2021). There are multiple polymorphisms in the *TAW1* promoter associated with its increased expression, and this allele was frequently found in aus accessions.

Inactivation of CK catalyzed by cytokinin oxidases is a key to controlling activities of this hormone in various cellular processes including shoot stem cell proliferation. Ashikari and colleagues firstly reported a QTL responsible for increased panicle branching in high-yielding cultivars (Ashikari *et al.* 2005). The causal gene, *Gn1a*, is *OsCKX2* with reduced expression and thereby enhancing the accumulation of CK in developing panicles (Ashikari *et al.* 2005). Subsequent studies revealed controlling mechanisms of *CKX* expression. A zinc-finger transcription factor DROUGHT AND SALT TOLERANCE (DST) directly activates the expression of *CKX*s including *OsCKX2* (Li *et al.* 2013). A dominant-negative allele of *DST* (*DST^reg1^*) lacking its C-terminus interferes with its wild-type function leading to the down-regulation of *CKX*s and increased panicle branching. A recent study further showed that DST is phosphorylated and activated by the ERECTA-MAPK signaling cascade, suggesting the presence of unknown signal molecules to regulate CK levels through this cascade in rice inflorescences (Fig. 6C) (Guo *et al.* 2020).

### Axillary meristem pathway for branch meristem formation

The genetic modules for axillary meristem formation in tillers, consisting of *LAX1*, *LAX2* and *MOC1*, also play indispensable roles in panicle branch meristem formation (Fig. 6D) (see also the section “Tiller”) (Komatsu *et al.* 2003a, Li *et al.* 2003, Tabuchi *et al.* 2011). Although strong alleles of *lax1*, *lax2* and *moc1* single mutations already show a significant reduction of branching, their higher order mutants often completely fail to form axillary meristems not only in vegetative shoots but also in inflorescences, suggesting their essential but partially overlapping functions in axillary meristem formation throughout the life cycle (Oikawa and Kyozuka 2009, Tabuchi *et al.* 2011). As is the case in tillers, *LAX1* mRNA is expressed in the boundaries between apical meristems and branch (or axillary) meristems, and its protein moves into the developing axillary meristem to exert its full function possibly through physical interaction with LAX2 (Oikawa and Kyozuka 2009, Tabuchi *et al.* 2011).

### The phase transition from branch meristems to spikelet meristems

In the later step of panicle development, the branching activity in PBMs and SBMs becomes restricted and all branch meristems eventually obtain the SM identity (Fig. 6D). *FRIZZY PANICLE* (*FZP*), encoding an AP2/ERF family transcription factor, is essential for this transition. *FZP* is specifically expressed at the base of spikelet meristem and just above rudimentary glumes and represses additional meristem formation in the axils of rudimentary glumes (Komatsu *et al.* 2003b). In *fzp* mutants, recurrent formation of axillary meristems results in an excessive ramification of inflorescence without floral organ differentiation (Komatsu *et al.* 2001, 2003b). Several QTL studies for panicle branching revealed multiple cis-regulatory changes in the *FZP* locus. In *O. sativa*, 4 bp deletion in the promoter occurred and reduced the expression level of *FZP* compared to that in *O. rufipogon* (Huang *et al.* 2018). An independent study identified a tandem duplication of an 18 bp silencer element upstream of *FZP* which increased spikelet per panicle in aus accession (Bai *et al.* 2017). Sequence polymorphisms in 3ʹ UTR of *FZP* also reduce its mRNA accumulation and increase secondary branches (Fujishiro *et al.* 2018). Thus, these studies indicate that cis-regulatory mutations which reduced *FZP* expression have been utilized multiple times to improve the inflorescence branch architecture.

*SPIKE*, a *NAL1* allele from tropical japonica landrace, increases panicle branching (Fujita *et al.* 2013). The effect of *SPIKE* is pleiotropic: it increases the size of both source and sink organs including roots, leaves and stems. The *SPIKE* allele possesses three amino acid substitutions in its coding region and also has an increased transcript level compared to those in standard cultivars. Therefore, the modified activity of the NAL1 protein or its increased abundance or both causes these phenotypes. *NAL1* encodes a trypsin-like serine and cysteine protease. Recently, Huang and colleagues reported that NAL1 physically interacts with FZP and promotes its degradation (Huang *et al.* 2018). Thus, NAL1 may regulate panicle branching through the regulation of FZP protein abundance (Fig. 6D).

### Initiation and maintenance of the spikelet meristem

Concurrently with the phase transition through the *FZP* pathway, communication between the lateral organ primordia and meristems plays a critical role (Fig. 6D). *YABBY* genes are known as representative regulators involved in this communication. In *Arabidopsis*, multiple mutants of *FIL*-related *YABBY* genes (*FIL*, *YABBY2*, *YABBY3*, and *YABBY5*) displayed defects not only in lateral organs such as leaves and floral organs but also in the meristem activity (Goldshmidt *et al.* 2008, Kumaran *et al.* 2002, Lugassi *et al.* 2010, Sarojam *et al.* 2010, Sawa *et al.* 1999, Siegfried *et al.* 1999, Stahle *et al.* 2009). These *YABBY* genes were expressed in lateral organs, but not in the meristems (Goldshmidt *et al.* 2008, Sarojam *et al.* 2010, Sawa *et al.* 1999, Siegfried *et al.* 1999). Furthermore, the FIL and YABBY3 proteins were demonstrated not to move between tissues (Goldshmidt *et al.* 2008). Therefore, the *YABBY* genes are likely involved in producing a putative signal that communicates from the lateral organs to the meristems. This assumption was supported by the result that the non-cell autonomous action of the *YABBY* genes was mediated in part by the activity of *LATERAL SUPPRESSOR*, which encodes a member of the GRAS family proteins and is expressed in the boundary region between the lateral organ primordia and meristems (Goldshmidt *et al.* 2008).

In rice, the function of three *TONGARI-BOUSHI* genes (*TOB1*, *TOB2*, *TOB3*), members of the *FIL* clade of the *YABBY* gene family, are closely associated with spikelet development (Fig. 6D). *TOB1* (also called *OsYABBY5*) was originally identified as the gene responsible for the maintenance of the meristem activity and lateral organ differentiation in spikelet development, since a loss-of-function mutant of *TOB1* showed premature termination of the spikelet meristem in addition to pleiotropic abnormalities in spikelet organs (Tanaka *et al.* 2012a, 2012b). *TOB1* was expressed exclusively in organ primordia, indicating that *TOB1* acts non-cell autonomously to maintain the proper activity of the spikelet meristem. Simultaneous knockdown of *TOB2* and *TOB3* (also called *OsYABBY4* and *OsYABBY3*, respectively) in the *tob1* mutant results in naked inflorescence branches without spikelets (Tanaka *et al.* 2017). Consistently, in the knockdown plant, spikelet meristems were lost in the axil of bracts during the early stages of inflorescence development. The three *TOB* genes were expressed in bract primordia, but not in the meristem, as expected. Thus, the non-cell autonomous action of the *TOB* genes is also required for the initiation of the spikelet meristem (Fig. 6D).

### Effect of flowering pathway genes on inflorescence development

The flowering pathway is also known to affect inflorescence architecture. *Ghd7* (for grain number, plant height and heading date), a *CONSTANCE-like* gene, repress flowering-promoting gene *Early heading date 1* (*Ehd1*) and *Hd3a* and thereby delays reproductive transition under the long-day condition. Thus, the prolonged vegetative phase in presence of functional *Ghd7* might result in a larger body size including culms and inflorescences (Xue *et al.* 2008). However, Endo-Higashi and Izawa proposed that the effect of flowering regulators on inflorescence development could be uncoupled with flowering time (Endo-Higashi and Izawa 2011). Using combinations of day length with the presence/absence of functional *Hd1* and *Ehd1* transgenes, they set up a condition in which each genotype flowered simultaneously. In this experiment, functional *Hd1* and *Ehd1* significantly reduced spikelet number per panicle, indicating that these flowering regulators affect inflorescence development independent of the duration of vegetative growth (Endo-Higashi and Izawa 2011). Another recent study revealed *MOTHER OF FT AND TFL1* (*OsMFT1*) as a possible mediator in this process. *Ghd7* activates *OsMFT1* expression, and in turn, *OsMFT1* represses *Ehd1*. *OsMFT1* also promotes panicle branching by repressing *FZP* and *SEP* subfamily of *MADS-box* genes (Song *et al.* 2018). These studies provide a possible explanation of how the flowering regulators modify inflorescence development (Fig. 6D).

### Regulation of panicle branch angles

The branch angle to the main axis (rachis) is another important trait in inflorescence because it is considered to affect the loss of grains before and during harvesting (Ishii *et al.* 2013). In domesticated cultivars, this angle is quite small causing the closed panicle advantageous in seed retention and harvesting. Wild species commonly have open panicles with a large branch angle due to the growth of pulvinus at the base of branches. This phenotype is controlled by a single locus called *SPR3*. Ishii and colleagues narrowed down the causal locus into a 9.3 kb region upstream of *OsLG1*. The allele from *O. rufipogon*, which is responsible for the open panicle phenotype, expresses *OsLG1* at higher levels compared to *O. sativa*, leading to the growth of pulvinus. The reduced nucleotide diversity in this region in cultivars suggested that the closed panicle phenotype linked to this locus was a trait selected during rice domestication (Ishii *et al.* 2013). Ishikawa and colleagues recently reported that a novel QTL *qSH3*, which encodes a YABBY transcription factor OsYABBY2, contributed to reducing seed shattering during domestication (Ishikawa *et al.* 2022). Interestingly, *qSH3* and another seed-shattering gene *sh4* (Li *et al.* 2006) are not sufficient to increase the harvesting index in wild rice background. The combination of these two genes with *SPR3*, however, significantly increased yield, suggesting that the closed panicle architecture had played a particularly important role during domestication (Ishikawa *et al.* 2022).

## Root

**Fig. 7.**
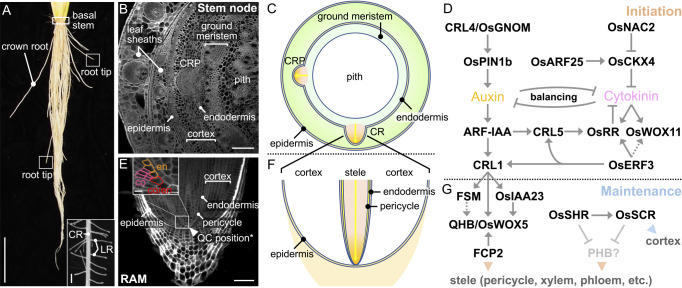
Fibrous root system of rice and the mechanisms of crown root initiation and maintenance. (A) A photograph illustrating the fibrous root system of rice. The inset photograph shows the lateral root (LR) that emerged from the crown root (CR). The scale bars are 5 cm and 100 μm (inset graph). (B) A tissue section of the stem node and the emerging crown root primordia (CRP). The scale bar is 50 μm. (C) A scheme of the stem node and the crown root (primordia). The yellow arrows indicate the auxin flow. (D) Auxin- and cytokinin-dependent regulatory networks during crown root initiation. Protein-protein interaction between OsERF3 and OsWOX11 might be not required for crown root initiation. (E) A tissue section of the apical part of the crown root. The inset graph shows the asymmetric cell divisions of cortex/endodermis initial cells (co/en) and cortical (co) and endodermal (en) cells. The scale bars are 50 μm and 5 μm (inset graph). The exact number of QC cells is controversial. (F) A scheme of the root apical meristem. The yellow arrow indicates the auxin flow. (G) Regulatory pathways of crown root maintenance. The dotted arrow indicates a lack of strong evidence which supports the interaction. PHB function is according to the analogy of *Arabidopsis*.

Rice has a fibrous root system typical of monocots consisting of a single seminal root, multiple crown roots and lateral roots (Figs. 1A, 7A) (Itoh *et al.* 2005). Unlike eudicots having a tap root system, the contribution of seminal roots to water and nutrient acquisition is limited in monocots (Viana *et al.* 2022). Accordingly, we focus on the developmental mechanisms of crown roots and lateral roots, both generated from the secondary RAMs during post-embryonic development. The fibrous root systems achieve larger spatial distribution than the tap root systems which rely on a single primary root (Yang *et al.* 2015). Moreover, they can have an advantage in the context of environmental adaptation: in some cases, monocots reconstruct most root systems in response to deleterious stress conditions, such as severe drought and soil flooding (Drew 1997, Sebastian *et al.* 2016). Even though the developmental mechanisms of monocot roots have been studied based on the analogy of *Arabidopsis* (Coudert *et al.* 2010, Hochholdinger *et al.* 2004), recent studies have revealed various aspects of monocot-specific root development.

### Crown root formation

#### Initiation of crown root primordia

Crown roots are shoot-born adventitious roots, post-embryonically emerging from the basal nodes (Fig. 7A–7C) (Coudert *et al.* 2010). Crown root initiation is the process to establish the apical meristem organization. The establishment of crown roots starts with an initial cell of crown root primordium which is generated by the periclinal division of the innermost ground meristem (Fig. 7B, 7C) (Itoh *et al.* 2005). The crown-root initial cells are then divided anticlinally and periclinally to organize the fundamental structure required for root function (Itoh *et al.* 2005, Viana *et al.* 2022). The cell types established at first are epidermis–endodermis, stele (central cylinder) and root cap (initials), and then the cortical cells are generated by the sequential periclinal (asymmetric) divisions of the cortex/endoderm initial cells (Fig. 7E) (Itoh *et al.* 2005, Kamiya *et al.* 2003a). Subsequently, several xylem vessels are formed in the stele, and then the crown root primordium vascularly connects to the ground tissue in the basal node (Fig. 7B) (Itoh *et al.* 2005, Viana *et al.* 2022).

#### Roles of auxin in crown root initiation

Auxin gradients at the vascular bundles and ground meristem cells are key for the initiation of crown root primordium (Fig. 7D) (Benkova *et al.* 2003), where PIN1-mediated auxin transport is controlled by the CROWN ROOTLESS 4 (CRL4) (Kitomi *et al.* 2008), a homologue of Arabidopsis GUANINE-NUCLEOTIDE EXCHANGE FACTOR (GNOM) having a role in the endosomal recycling of PIN1 (Geldner *et al.* 2003). In the *crl4* mutant, the single nucleotide substitution in the *OsGNOM* gene results in the premature stop codon upstream of the conserved domain, thereby suppressing the crown root initiation (Kitomi *et al.* 2008). This phenotype is confirmed by the additional four independent alleles of rice *gnom1* mutants (Liu *et al.* 2009). Indeed, the transcripts of *CRL4* and *OsPIN1b* are simultaneously detected in the ground meristem cells (Kitomi *et al.* 2008, Xu *et al.* 2005). The *DR5:GUS* staining, which is a reliable marker of auxin response, is detected at the ground meristem cells in the basal nodes of the wild-type rice, whereas the staining is faint and diffused in the *crl4* mutant (Kitomi *et al.* 2008). These suggest that OsPIN1b-mediated auxin transport controlled by CRL4/OsGNOM is indispensable for crown root initiation in rice.

In the presence of auxin, a protein complex composed of an auxin receptor TRANSPORT INHIBITOR RESPONSE 1 (TIR1) and AUXIN SIGNALING F-BOX (AFB) proteins, which are F-box proteins compose the SKP1-CUL1-F-box protein (SCF) ubiquitin ligase complexes, targets AUXIN/INDOLE-3-ACETIC ACID (AUX/IAA) proteins for ubiquitylation and degradation (Dharmasiri *et al.* 2005, Gray *et al.* 2001). IAA is a repressor of the function of ARF transcription factors (Tiwari *et al.* 2004), and thus the auxin-dependent de-repression of ARF by the IAA degradation is fundamental for auxin signal transduction (Fig. 7D) (Chapman and Estelle 2009). *CRL1*, which encodes an AS2/LOB transcription factor, OsLBD3-2, is a direct target of ARF transcription factor(s) (Inukai *et al.* 2005). Single amino acid substitution in the N-terminal region of CRL1 in the *crown rootless1* (*crl1*) mutant, and the frameshift at the middle part of CRL1 in the *Adventitious rootless1* (*arl1*) mutant, both result in the severe defect of crown root formation (Inukai *et al.* 2005, Liu *et al.* 2005). These suggest that CRL1 functions downstream of CRL4- and PIN1-dependent auxin distribution.

Recently, it has been demonstrated that the *OsSPL3* transcription factor, which is the causal gene for the *lower crown root number1* (*lcrn1*) mutant, is involved in the crown root initiation (Shao *et al.* 2019). The *lcrn1* mutant, in which a point mutation prevents the miR156-directed cleavage of *OsSPL3* transcripts, produces fewer crown root primordia than its background wild type (Shao *et al.* 2019). ChIP-seq and RNA-seq analyses in the basal nodes of the *lcrn1* mutant identified *OsMADS50* as a direct target of OsSPL3 (Shao *et al.* 2019). The *OsARF19* and *OsPIN2* expressions are suppressed in the *lcrn1* and *OsMADS50*-overexpressors, suggesting that the *miR156-OsSPL3-OsMADS50* module contributes to the regulation of auxin transport and signaling during crown root initiation (Shao *et al.* 2019). However, it remains to be clarified how these mechanisms are integrated into the CRL4-CRL1-dependent crown root initiation in rice.

#### Auxin and cytokinin interaction in crown root development

CK is another phytohormone having a key role in crown root development (Fig. 7D). The *OsWOX11* is expressed in the meristematic region of the basal nodes which include the emerging crown roots (Zhao *et al.* 2009). Loss of function mutation in *OsWOX11* caused by T-DNA insertion results in little or no crown root production, whereas the overexpressors of *OsWOX11* produce many shoot-borne roots at every node (Zhao *et al.* 2009). Interestingly, the *OsWOX11* expression is induced not only by an exogenously supplied auxin (IAA) but also by a CK, 6-benzylaminopurine (6-BA) (Zhao *et al.* 2009). Another line of evidence supporting the roles of CK in crown root initiation is the reduction of crown root number in the *crl5* mutant, which has a premature stop codon in a gene encoding ERF transcription factor (Kitomi *et al.* 2011). Among the 13 type-A RESPONSE REGULATORs (RRs), which act as negative regulators of CK signaling (Ito and Kurata 2006, To and Kieber 2008), only the *OsRR1* and *OsRR2* expressions are activated by the *CRL5* overexpression (Fig. 7D) (Kitomi *et al.* 2011). The ectopic expression of *OsRR1* through the control of the *CRL5* promoter partly restores the crown root formation of the *crl5* mutant, indicating that repression of CK signaling by OsRR1 (and/or OsRR2) stimulates the crown root initiation in rice (Fig. 7D) (Kitomi *et al.* 2011).

Knockdown of a gene encoding the OsERF3 transcription factor in the *wox11* mutant shows a further reduction of the number of crown root primordia, whereas the increased expression of *OsERF3* partially complements *wox11* mutants, suggesting that OsWOX11 and OsERF3 cooperatively function in the crown root initiation (Fig. 7D) (Zhao *et al.* 2015). Interestingly, overexpression and knockdown of *OsERF3* result in the induction and suppression of the *CRL1*, *CRL4* and *CRL5* as well as *OsRR1* and *OsRR2* expression, respectively (Zhao *et al.* 2015). These suggest that OsERF3 stimulates crown root initiation through the induction of the *CRL* and type-A *RR* genes. The *OsERF3* expression is induced by the exogenous treatments of auxin and CK as is the case with *OsWOX11*, further suggesting that the OsWOX11- and OsERF3-mediated balancing of auxin and CK signals contribute to the crown root initiation in rice (Zhao *et al.* 2009, 2015).

Crown root number increases in a rice dominant mutant, *root enhancer1* (*ren1*-*D*), in which the transcription level of *OsCKX4* is increased by the effect of the cauliflower mosaic virus *35S* enhancer (Gao *et al.* 2014). *OsCKX4* is predominantly expressed in the region where crown root primordia initiate (Gao *et al.* 2014). As the *OsCKX4* promoter is a direct target of OsARF25 and the type-A RRs, auxin and CK coordinately regulate the *OsCKX4* expression (Gao *et al.* 2014). The *OsCKX4* expression in roots is induced by 6-BA and IAA within 30 min, but this induction is suppressed within 2 h after the initiation of the treatments (Gao *et al.* 2014). The recent finding has shown that the NAC (NAM, ATAF and CUC) transcription factor (NAC2), the expression of which is stimulated by 6-BA and IAA, suppresses the *OsCKX4* expression (Mao *et al.* 2020). These suggest that the feedback and balance control of auxin and CK signals is essential for the proper crown root initiation in rice.

It should be noted that the *OsWOX11* expression is remarkable after the primordia initiation in the emerging crown root primordia (Zhao *et al.* 2009), suggesting that OsWOX11 predominantly functions in crown root maintenance. In emerging crown roots, OsWOX11 interacts with OsERF3 and cooperatively represses *OsRR2* expression to increase CK signaling and stimulate crown root elongation (Zhao *et al.* 2015). Further studies are required to clearly distinguish the auxin-CK interaction mechanisms in crown root initiation and subsequent developmental processes.

#### Mechanism of radial patterning of crown root and its maintenance

Crown root maintenance is the continuous cell division of the fully established apical meristems (Fig. 7E, 7F). In this process, the QC plays a pivotal role to maintain the activities of surrounding stem cells (Strotmann and Stahl 2021). However, the number of QC cells is still controversial in rice. Histological observations and the expression of the GRAS family transcription factor SCARECROW (SCR) suggest that the number of QC cells in rice is very small as in *Arabidopsis* (around four) (Ni *et al.* 2014). However, more than four mitotically inactive cells are identified in rice RAMs concomitantly with the broader *QUISCENT-CENTER-SPECIFIC HOMEOBOX (QHB)/OsWOX5* promoter activity (Ni *et al.* 2014). Although the QC identity of these cells remains to be determined, a higher number of QC cells might be required for the maintenance of the larger RAMs in rice and other cereals (Strotmann and Stahl 2021).

CRL1 functions in crown root maintenance through the control of QHB/OsWOX5 (Fig. 7G) (Inukai *et al.* 2005, Kamiya *et al.* 2003b). OsIAA23 functions in the maintenance of QC identity, also through the control of QHB/OsWOX5, possibly downstream of CRL1 (Jun *et al.* 2011). In addition, CLE-WOX signaling maintains root stem cell niche in rice as is the case in *Arabidopsis* (Chu *et al.* 2013). The overexpression of *FCP2* (also known as *CLE50*) or the exogenous application of a synthetic FCP2 peptide (FCP2p) terminates root apical meristem activity and impairs late metaxylem formation (Chu *et al.* 2013). Moreover, FCP2p suppresses the *QHB*/*OsWOX5* expression in the QC and late metaxylem cells (Chu *et al.* 2013), indicating that FCP2-QHB/OsWOX5 signaling controls root cell proliferation (Fig. 7G). Another gene associated with the QHB/WOX5 function is *FLATTENED SHOOT MERISTEM* (*FSM*) which encodes the p150 subunit of chromatin assembly factor-1 (Abe *et al.* 2008). As the *Tos17* insertion mutant of *FSM* shows the disruption of the radial patterning of the crown roots, FSM functions in the maintenance of the tissue organization of crown roots (Abe *et al.* 2008). Transactivation assays using rice protoplast confirmed that CRL1 activates *FSM* and *QHB* promoters, supporting that CRL1 is an initiator of crown root maintenance (Fig. 7G) (Lavarenne *et al.* 2019).

The radial pattern of the crown roots, composed of three concentric cell layers, the epidermis, cortex and stele, is determined by the fine-tuning of asymmetric cell divisions in the apical meristems (Fig. 7E, 7F) (Itoh *et al.* 2005). The primary root of *Arabidopsis* has a simple anatomical structure and forms a single cortex layer, when the endodermis (a part of the cortex) and middle cortex (generated only in the late stage) are not considered (Dolan *et al.* 1993, Lux *et al.* 2004, Paquette and Benfey 2005), whereas in rice, the cortex/endoderm initial cells periclinally divide several times to produce multiple cortical cell layers (Fig. 7E) (Kamiya *et al.* 2003a). In *Arabidopsis*, a GRAS family transcription factor, SHORT-ROOT (SHR) is expressed in the stele and functions as a trigger of the asymmetric cell divisions in the stele (Cui *et al.* 2007). SHR moves into the adjacent cell layer and stimulates the expression of *SCR*, which has a role in the asymmetric divisions of the cortex/endoderm initial cells, to specify the endodermis cell layer (Cui *et al.* 2007, Wu *et al.* 2014). SCR simultaneously functions in the restriction of the radial extent of SHR movement by yet unknown mechanisms (Wu *et al.* 2014). Although this local feedback mechanism is essential for the radial patterning of primary roots in *Arabidopsis* (Cui *et al.* 2007, Wu *et al.* 2014), recent findings in rice, maize and barrel medic (*Medicago truncatula*) cast doubt on the conservation of this mechanism in most plant species.

Interestingly, the expression of rice *OsSHR*s fused with *AtSHR* promoter results in the formation of multiple cortical cell layers in *Arabidopsis* roots due to the higher mobility of OsSHR proteins compared to AtSHR (Wu *et al.* 2014). In addition, overexpression of *OsSHR* results in the excess numbers of cortex-cell layers in rice (Henry *et al.* 2017). Recently, it has been demonstrated that maize ZmSHR protein moves at least eight cell layers into the cortex, and this leads to the sequential divisions of the cortical cells to develop multiple cortical cell layers (Ortiz-Ramírez *et al.* 2021). Furthermore, MtSHR1 and MtSHR2 of *M. truncatula*, which are transcribed in the stele, also translocate into the multi-layered cortex to promote cortical cell division and nodule formation (Dong *et al.* 2021). These studies strongly suggest that differences in the radial extent of SHR movement generate variations in the number of cortical cell layers in plants.

In *Arabidopsis*, SCR and SHR indirectly suppress the gene encoding HD-ZIP III transcription factor, PHABULOSA (PHB) (Carlsbecker *et al.* 2010, Miyashima *et al.* 2011). Despite the importance of the PHB function in stele development, such as xylem specification and pericycle differentiation in *Arabidopsis* (Carlsbecker *et al.* 2010, Miyashima *et al.* 2011), the function of PHB or homologous HD-ZIP III proteins in rice remains to be characterized. Recently, cell type-specific mRNA purification by translating ribosome affinity purification (TRAP) in rice roots revealed that two PHB-like HD-ZIPIII homologues are indeed expressed in the developing xylem cells, which are marked by the *QHB/OsWOX5* expression (Reynoso *et al.* 2022). Thus, the mechanisms of root radial patterning and its maintenance could be conserved between *Arabidopsis* and rice with some exceptions to determine the complex organization of the rice crown root.

### Crown root growth

#### Regulation of crown root outgrowth

**Fig. 8.**
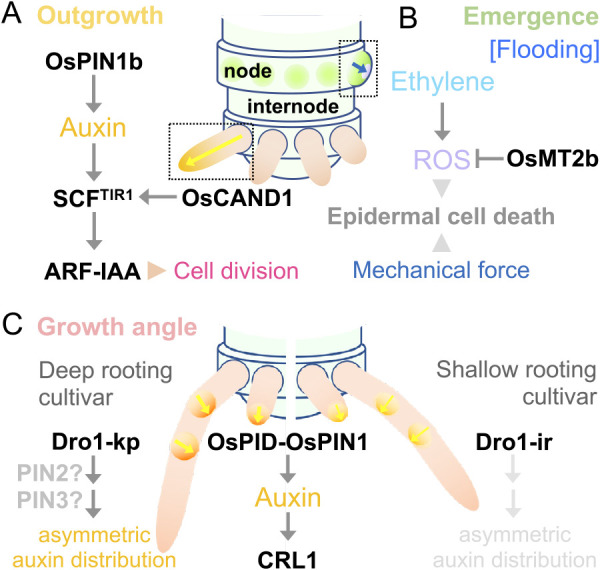
Regulators of crown root outgrowth, emergence and growth angle. (A) A scheme of the crown root outgrowth and its regulators. The yellow arrow indicates the auxin flow. (B) A scheme of the crown root emergence under flooding and its regulators. The blue arrow indicates the mechanical force originating from crown root outgrowth. (C) A scheme of the crown root growth angle in deep rooting and shallow rooting cultivars, and the regulators of root gravitropic response. The yellow arrows indicate the auxin flows and the thickness of the arrows indicates the abundance of auxin flow. PIN2 and PIN3 functions are according to the analogies of *Arabidopsis*.

Crown root outgrowth is a wide view of crown root maintenance being required for the continuous elongation of the individual crown roots (Fig. 8A). Although the treatments of auxin flux inhibitors decrease the numbers of emerged crown roots (Zhou *et al.* 2003), these observations include the suppression of crown root initiation. The first report clearly showing the involvement of auxin in the crown root outgrowth is the study using the *OsPIN1b* RNAi knockdown lines (Xu *et al.* 2005). In the young seedlings, crown root primordia are detected at the first node of the wild type and *OsPIN1b* knockdown lines, however, their emergences are detected only in the wild type (Xu *et al.* 2005). As the crown root primordia in the *OsPIN1b* knockdown lines arrest at the stage where the fundamental organization of apical meristems is established (Xu *et al.* 2005), PIN1-dependent auxin transport is required for the outgrowth of crown roots. ENHANCER OF TIR1 AUXIN RESISTANCE2 (ETA2)/CULLIN-ASSOCIATED AND NEDDYLATION-DISSOCIATED1 (CAND1) promotes the assembly of SCF^TIR1^ auxin receptor complex (Fig. 8A) (Chuang *et al.* 2004). The rice *cand1* mutant, in which the single nucleotide substitution causes a premature stop codon in *OsCAND1*, forms the same number of crown root primordia as the wild type, whereas the *cand1* mutant fails to emerge any crown roots (Wang *et al.* 2011). The transcription level of the G2/M transition indicator *CYCLINB1;1* significantly decreases in the crown root primordia of the *cand1* mutant, suggesting that the auxin perception by the SCF^TIR1^ is required for the stimulation of cell divisions during crown root outgrowth (Wang *et al.* 2011).

#### Regulation of crown root emergence

Crown root emergence is accompanied by the death of the nodal epidermal cells which support the penetration of crown roots from inside to outside of the nodes (Fig. 8B). Mechanisms of crown root emergence from the surface of nodes have been studied in the context of flood adaptation of deepwater rice (Lorbiecke and Sauter 1999, Suge 1985). Epidermal cell death, which is confined to the nodal epidermal cells covering the tip of crown root primordia, facilitates the emergence of crown roots (Mergemann and Sauter 2000). Ethylene-induced accumulation of reactive oxygen species (ROS) triggers programmed cell death (PCD) in the epidermis (Steffens and Sauter 2009). The knockdown of a gene encoding ROS scavenging protein, METALLOTHIONEIN2b (OsMT2b), accelerates the epidermal cell death, supporting that ROS accumulation in the epidermis is essential for the crown root emergence under flooding (Fig. 8B) (Steffens and Sauter 2009). On the other hand, ethylene-ROS signaling is also required for crown root outgrowth, and this generates a mechanical force stimulating PCD in the epidermal cells (Steffens *et al.* 2012). The artificial mechanical pressure induces the ectopic PCD in the epidermis even under non-flooding conditions (Steffens *et al.* 2012), suggesting that these mechanisms are not exclusive to the flood adaptation of deepwater rice and possibly underly in the normal development of lowland rice.

#### Regulation of crown root growth angle

Regulation of the root growth angle is somewhat analogous to that of the tiller and panicle branch angles with respect to the auxin-dependent gravitropic responses (Fig. 8C) (Li *et al.* 2019). The CRISPR/Cas9-based *pin1a pin1b* double knockout rice shows increased root growth angle, tiller angle and panicle branch angle, whereby the organs grow in more horizontal directions (Li *et al.* 2019). Reduced gravitropism in crown roots is frequently observed in the rice mutants defective in auxin signaling (Inukai *et al.* 2005, Kitomi *et al.* 2011, Liu *et al.* 2009), suggesting that auxin is also involved in regulating the root growth angle in rice. For instance, the response of roots to the gravity change is impaired in the *crl1* mutant (Inukai *et al.* 2005). PINOID (PID), a serine-threonine protein kinase, phosphorylates PIN proteins and stimulates apical-to-basal shifts in PIN localization of *Arabidopsis* (Friml *et al.* 2004). The gravitropism in the seminal roots was strongly reduced by the overexpression of a gene encoding rice ortholog of PID (Morita and Kyozuka 2007). Moreover, polar auxin transport inhibitor NPA reduces the root gravitropic response (Morita and Kyozuka 2007).

The growth angle of crown roots is a major determinant of root system architecture in rice (Uga *et al.* 2015). *DEEPER ROOTING 1* (*DRO1*) was identified as a causal gene for the QTL of root growth angle in rice (Uga *et al.* 2013). At that time, DRO1 was characterized as a plasma-membrane localized protein with unknown function due to the lack of sequence conservation among homologous genes (Uga *et al.* 2013). However, later it was classified into a different clade of IGT family proteins from LAZY1 and TAC1 (Hollender and Dardick 2015) (for the IGT family proteins, see the section “Tiller”). Although the LAZY1 is essential for shoot-gravitropism controls, the seminal roots of rice *lazy1* mutants do not show any abnormal gravitropic response (Yoshihara and Iino 2007). *DRO1* is detected in recombinant inbred lines (RILs) derived from a cross between the lowland rice cultivar ‘IR64’ with shallow rooting and the upland rice cultivar ‘Kinandang Patong (KP)’ with deep rooting (Uga *et al.* 2011). IR64 and KP respectively have non-functional and functional alleles of *DRO1*, indicating that DRO1 reduces the root growth angle to establish the deep rooting system in KP (Uga *et al.* 2013). *DRO1* transcripts accumulated in the upper side of the elongation zone during the rotation of the horizontal roots of the *Dro1* near-isogenic line (NIL), which has the functional *DRO1* gene in the IR64 background, indicating that DRO1 enhances the elongation in the upper side of the crown roots, and stimulates downward root bending (Fig. 8C) (Uga *et al.* 2013).

Root gravitropism is regulated by the asymmetric auxin distribution in *Arabidopsis*, where PIN3 and PIN7 polarize to the lower side of columella cells to mediate auxin transport to the gravity direction (Han *et al.* 2021). Moreover, PIN2 is highly expressed on the lower side of the rotated primary roots and stimulates the auxin transport from the columella cells to the elongation zone especially on the lower side, where a high level of auxin inhibits cell elongation leading to the downward bending of the primary roots (Han *et al.* 2021). Asymmetric auxin distribution in response to gravistimulation has been also demonstrated in rice (Lucob-Agustin *et al.* 2020), and the rice *pin2* mutant shows curly root phenotypes with a reduced gravitropic response (Inahashi *et al.* 2018). Taking these into account, *DRO1* transcription in the lower side of bending roots could be suppressed by the high concentration of auxin, and this asymmetry of *DRO1* expression is required for the efficient gravitropic response in the deep rooting upland cultivars (Fig. 8C) (Uga *et al.* 2013).

### Crown root anatomy

#### Anatomical structure of the crown root

**Fig. 9.**
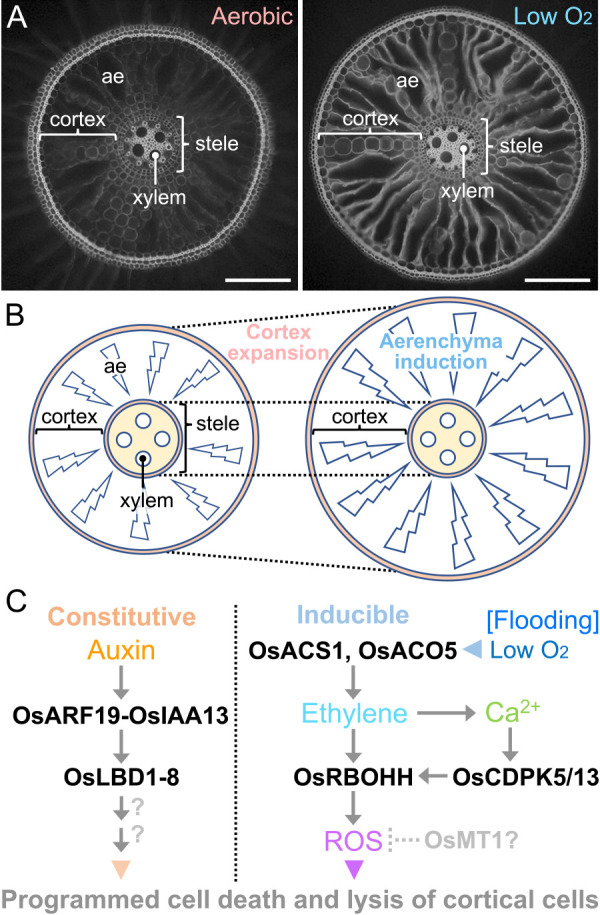
Mechanisms of constitutive and inducible aerenchyma formation. (A) Cross-sections of the crown roots of rice seedlings grown under aerobic and low-oxygen conditions (low O_2_). The scale bars are 100 μm. ae; aerenchyma. (B) A scheme of the anatomical structures of the crown roots. The cortex is expanded and aerenchyma is induced under lox-oxygen conditions. ae; aerenchyma. (C) Regulatory pathways of constitutive aerenchyma formation under aerobic conditions and inducible aerenchyma formation under flooding (low-oxygen) conditions. The involvement of OsMT1s in inducible aerenchyma formation is postulated based on the reactive oxygen species (ROS) scavenging activity of the OsMT1 proteins and their expression patterns.

Roots mainly consist of three concentric cell layers, the epidermis, cortex and stele (Fig. 7E, 7F) (Coudert *et al.* 2010, Dolan *et al.* 1993). The stele contains xylem vessels having a role in the water and nutrients transport to the aerial tissues, and phloem tissue has a role in the transport of photosynthate from the aerial tissues to the root tips (Petricka *et al.* 2012). Most plant species have multiple cortical cell layers, even though they require more energy to maintain many cortical cells (Yamauchi *et al.* 2021a). As cortical cells are a reservoir of water and nutrients, roots with multiple cortical cell layers may have the advantage to enlarge root systems and achieve large shoot biomass. Lysigenous aerenchyma is an interconnected gas space created by the PCD and subsequent lysis of the cortical cells (Fig. 9A, 9B) (Colmer 2003). Aerenchyma enhances oxygen diffusion within plants under flooding and reduces (adjusts) carbon and nutrient costs for the maintenance of root cells under flooding (Colmer 2003). Under drought and nutrient deficiency, aerenchyma contributes to reducing the metabolic costs for the maintenance of root cortical cells and stimulates root elongation for soil water and nutrient exploration (Lynch 2018). The primary root of *Arabidopsis*, which comprises a single or double cortical cell layer (Petricka *et al.* 2012), has no lysigenous aerenchyma, supporting the idea that levels of aerenchyma formation determine the balance between the benefits and costs for plant survival in environmental constraints. Crown roots of rice developmentary form aerenchyma under the condition without any stressors, and further induces its formation in response to flooding (Fig. 9A, 9B) (Yamauchi and Nakazono 2022a). Former and the latter are designated as constitutive and inducible aerenchyma formations, respectively (Colmer and Voesenek 2009).

#### Mechanisms of aerenchyma formation

Root cells are generated from the apical part of the roots, and thus their ages gradually progress toward the basal parts (Coudert *et al.* 2010, Dolan *et al.* 1993). The longitudinal organization of root cells in rice is similar to that in *Arabidopsis* except for the complexity of the structural organization, which comprise meristematic, elongation, and differentiation zones from the apical part to the basal part (Coudert *et al.* 2010, Petricka *et al.* 2012). In rice roots, the longitudinal position where aerenchyma is first detected is closely associated with the position of the lateral root initiation (Kono 1968), i.e., in the differentiation zone (Hochholdinger *et al.* 2004). Lateral root number severely decreases in the rice *iaa13* mutant, in which dominant negative OsIAA13 prevents auxin signaling (Kitomi *et al.* 2012). Interestingly, constitutive aerenchyma formation is also reduced in *iaa13*, and the reduction is confirmed by the treatment with a polar auxin transport inhibitor, *N*-1-naphthylphthalamic acid (NPA) (Yamauchi *et al.* 2019b). OsIAA13 physically interacts with OsARF19, which binds to the promoter of *OsLBD1-8* (Fig. 9C) (Yamauchi *et al.* 2019b). The complementation of the *iaa13* mutant by the ectopic expression of *OsLBD1-8* fused with the *OsIAA13* promoter, partially restores aerenchyma and lateral root formations (Yamauchi *et al.* 2019b), suggesting that the OsARF19- and OsIAA13-mediated transcriptional control of *OsLBD1-8* determines the pattern of root cell differentiation. Despite forming lateral roots, roots of upland plants, such as maize and wheat, generally form limited constitutive aerenchyma (Colmer and Voesenek 2009), suggesting that rice has integrated the system of OsARF19- and OsIAA13-mediated aerenchyma formation into the normal root development during evolution.

#### Regulation of anatomical plasticity of crown root

Optimal anatomical structures to adapt to various soil water levels are determined by the field evaluation of Poaceae species (Yamauchi *et al.* 2021b). The species having larger stele, in which larger xylem vessels support efficient water transport, and those having larger cortex, in which greater aerenchyma contributes to the efficient oxygen diffusion, are more adaptive to drought and flooding, respectively (Yamauchi *et al.* 2021b). Due to the benefit of aerenchyma to provide oxygen and reduce metabolic costs (Colmer 2003, Lynch 2018), the species found in both drought and flooding conditions have large proportions of aerenchyma in their roots (Yamauchi *et al.* 2021b). Interestingly, rice remarkably increases cortex area (size and number of the cortical cells) in response to oxygen deficiency, and this leads to the increase of oxygen diffusion through aerenchyma in the crown roots (Fig. 9A, 9B) (Yamauchi *et al.* 2019a). Although OsSHR and OsSCR should be involved in the increase of cortical cell layers, such mechanisms remain largely unknown. As the timing of the increase in the cortical cell layers in the crown root primordia is well associated with the increase in the phloem number (Kawata and Harada 1977), compounds that are transported from the aerial tissues, i.e., the photosynthates and/or auxin might determine the number of cortical cell layers.

Aerenchyma formation in roots of rice and other upland species, such as maize and wheat, is induced in response to oxygen deficiency under soil flooding (Fig. 9A, 9B) (Colmer and Voesenek 2009, Yamauchi and Nakazono 2022a). Physical entrapment due to the restricted gas exchange enhances the accumulation of gaseous phytohormone ethylene under flooding (Sasidharan *et al.* 2018, Yamauchi *et al.* 2018). Moreover, the expression inductions of the genes encoding ethylene biosynthesis enzymes, 1-AMINOCYCLOPROPANE-1-CARBOXYLIC ACID (ACC) SYNTHASE 1 (OsACS1) and ACC OXIDASE 5 (OsACO5) further enhance ethylene accumulation (Fig. 9C) (Yamauchi *et al.* 2017). Ethylene stimulates the cortex-specific expression of the gene encoding RESPIRATORY BURST OXIDASE HOMOLOG H (OsRBOHH), which is a key enzyme in ROS production, converting oxygen (O_2_) to superoxide anion radical (O_2_^•–^) at the apoplast (Suzuki *et al.* 2011, Torres and Dangl 2005). Moreover, the CALCIUM-DEPENDENT PROTEIN KINASE 5/13 (OsCDPK5/13), which are also highly expressed in the cortex, phosphorylates OsRBOHH protein and stimulates the activity of O_2_^•–^ production, thereby inducing PCD of the cortical cells (Yamauchi *et al.* 2017). During the inducible aerenchyma formation, the expression levels of *OsRBOHH* and *OsCDPK5*/*13* somewhat increase in the outer part of crown roots (Yamauchi *et al.* 2017). The higher expression levels of the genes encoding ROS scavenging protein OsMT1s might cancel the ROS accumulation in the outer part of crown roots (Fig. 9C) (Yamauchi *et al.* 2018).

Modeling-based age-dependent analyses of the auxin-dependent (ethylene-independent) aerenchyma formation and ethylene-dependent aerenchyma formation dissect the patterns of each type of aerenchyma formation (Yamauchi and Nakazono 2022b). Ethylene-dependent aerenchyma formation is more rapid than auxin-dependent aerenchyma formation, and thus ethylene-dependent aerenchyma formation could contribute more greatly to the oxygen diffusion to the apical part of the crown roots at the onset of flooding (Yamauchi and Nakazono 2022b). On the other hand, ethylene biosynthesis requires oxygen, and thus auxin-dependent constitutive aerenchyma formation can support oxygen demands for ethylene production at the onset of flooding (Yamauchi *et al.* 2018). Although the fundamental hormonal controls of both types of aerenchyma formation have been demonstrated, the master regulators, such as transcription factors, remain undetermined (Yamauchi and Nakazono 2022a).

### Lateral root formation

#### Mechanisms of lateral root formation

Lateral roots enhance water and nutrient uptake from the soils by increasing of total surface areas of the root systems (Pélissier *et al.* 2021, Viana *et al.* 2022, Yu *et al.* 2016). As drought-tolerant rice genotypes usually have greater root length density, the levels of lateral root branching are essential for drought adaptation (Lucob-Agustin *et al.* 2021). In rice, long and thick lateral roots (L-type lateral roots) with a well-developed vasculature produce shorter and thinner secondary lateral roots (S-type lateral roots) that do not form further branches (Lucob-Agustin *et al.* 2021, Suralta *et al.* 2018). Thus, the L-type lateral roots more strongly contribute to the expansion of the lateral root distribution than the S-type lateral roots (Lucob-Agustin *et al.* 2021).

**Fig. 10.**
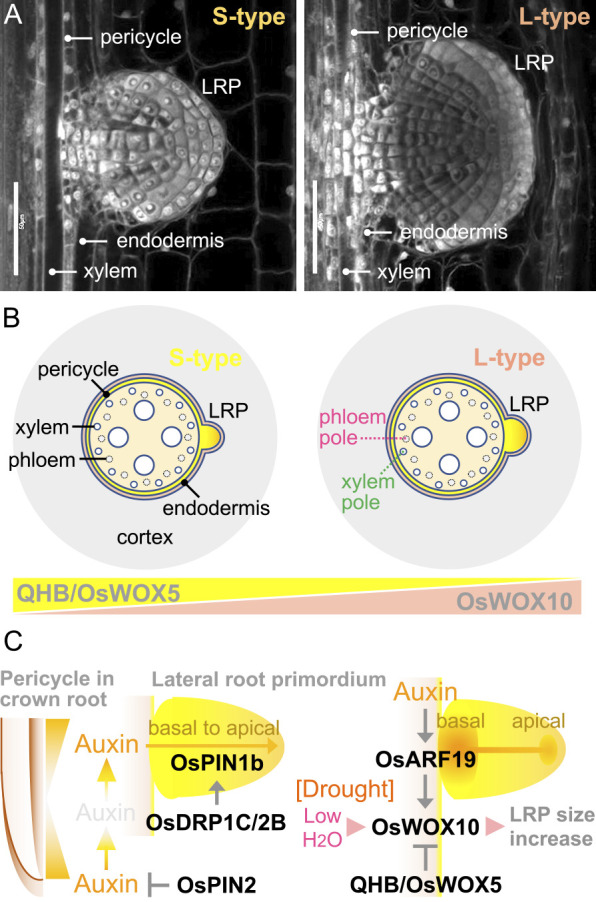
Regulation of S-type and L-type lateral root formations. (A) Tissue sections of the S-type and L-type lateral root primordia (LRP). The scale bars are 50 μm. (B) A scheme of the S-type and L-type lateral root primordia (LRP) formations and the key regulators of the size of lateral root primordia. (C) Molecular mechanisms that regulate LRP initiation in the pericycle of the crown roots and the auxin flux from the pericycle to the apical part of the LRP (left). Regulatory pathways that determine the size of LRP (right). Accumulation of auxin in the basal part of the LRP stimulates the increase of LRP size.

Lateral roots originated from the pericycle cells located in the differentiation zone of main roots, which includes primary (seminal) and shoot-borne adventitious (crown) roots (Figs. 7A, 10A) (Hochholdinger *et al.* 2004, Kono *et al.* 1972, Petricka *et al.* 2012). In *Arabidopsis*, the lateral root primordium initiates at pericycle cells located at the xylem poles, whereas it initiates at pericycle cells close to the phloem poles in rice crown roots (Fig. 10B) (Viana *et al.* 2022). Although the lateral root primordia originated from the pericycle cells in *Arabidopsis* (Dubrovsky *et al.* 2000), those in rice and maize originated from the pericycle cells and endodermal cells (Fig. 10B) (Hochholdinger *et al.* 2004, Yu *et al.* 2016).

In *Arabidopsis*, lateral roots initiate at the end of the elongation zone of the primary root, where the auxin level is much lower than that in the apical part of the root (Dubrovsky *et al.* 2011). This ‘auxin minimum’ is achieved by the CK-dependent stimulation of the genes encoding GRETCHEN HAGEN 3 (GH3) family proteins, which inactivate the auxin (IAA) through the conjugation with aspartic acid or glutamic acid (Fig. 10C) (Staswick *et al.* 2005), and the repression of the local PIN-dependent auxin transport (Di Mambro *et al.* 2017). The lateral root cap cells undergo cyclic cell death when they enter the elongation zone of the primary roots, which release auxin to the surrounding root tissue, providing a local auxin source that stimulates cyclic lateral root formation (Xuan *et al.* 2016). Although the detailed molecular mechanisms remain unclear, the local auxin gradient also contributes to the lateral root formation in the crown roots of rice (Yamauchi *et al.* 2019b). *OsPIN2* is expressed in the root epidermal cells and the outer cortex cells, which is analogous to the expression patterns of *PIN2* in *Arabidopsis* (Müller *et al.* 1998, Wang *et al.* 2018b), and regulates the redirection of auxin flux from downward to upward into the elongation zone (Chen *et al.* 2012). Retardation of the establishment of “auxin minimum” due to the disruption of *OsPIN2* function in the rice *pin2* mutant results in the increase of auxin level and reduction of lateral root number in the apical part of the crown roots (Fig. 10C) (Inahashi *et al.* 2018).

The fundamental mechanisms of lateral root initiation are common in *Arabidopsis* and rice, where ARF- and IAA-dependent auxin signaling plays a central role (Fukaki *et al.* 2002, Kitomi *et al.* 2012, Okushima *et al.* 2005, Zhu *et al.* 2012). The dominant-negative mutation of the *OsIAA11* or *OsIAA13* gene results in the severe reduction of lateral root numbers (Kitomi *et al.* 2012, Zhu *et al.* 2012). OsIAA13 physically interacts with OsARF19 *in vitro* and the OsARF19-mediated transcriptional regulation of *OsLBD1-8* at least partly contributes to the lateral root formation in rice (Yamauchi *et al.* 2019b). Interestingly, rice OsARF19 and OsIAA13 are closest to the major contributors to lateral root initiation in *Arabidopsis*, i.e., ARF7/19 and SOLITARY ROOT (SLR)/IAA14, respectively (Fukaki *et al.* 2002, Okushima *et al.* 2005).

T-DNA and *Tos17* insertion mutants of *OsARF11*, which is the closest homologue of *MONOPTEROS* (*MP*)/*ARF5* in *Arabidopsis*, show the reduction of lateral root numbers with the other pleiotropic phenotypes (Sims *et al.* 2021). As the homozygous *arf11* mutants showed low fertility or complete sterility, OsARF11 would function in early embryogenesis as is the case of MP/ARF5 (Schlereth *et al.* 2010, Sims *et al.* 2021). In *Arabidopsis*, the BODENLOS (BDL)/IAA12-MP/ARF5-dependent module functions in the later stage of lateral root development compared with the SLR/IAA14-ARF7/19-dependent lateral root initiation module (De Smet *et al.* 2010). The weak reduction of lateral root number in the *arf11* mutants (Sims *et al.* 2021) suggests that OsARF11 might function in the late stage of lateral root development. However, further studies are needed to demonstrate the analogous function of OsARF11 in lateral root development.

#### Mechanisms of S-type and L-type lateral root formation

The seminal and crown roots of rice form two types of lateral roots, that is, long and thick L-type lateral roots and short and thin S-type lateral roots, and the formation of either type is determined by the environmental cues (Lucob-Agustin *et al.* 2021, Suralta *et al.* 2018). L-type lateral roots can be stimulated by the surgical excision of parental root tips, which mimics the compensatory growth of lateral roots in response to the mechanical impedance of root growth under soil compaction (Kawai *et al.* 2017).

The seminal roots of a rice mutant having a premature stop codon in the *QHB*/*OsWOX5* gene produce more L-type lateral roots than its background wild type in response to the root tip excision (Kawai *et al.* 2022b). RNA-seq comparison between the L-type and S-type lateral root primordia reveals that the transcripts of *OsWOX6*, *OsWOX10* and *OsWOX11* are more abundant in the L-type lateral root primordia (Kawai *et al.* 2022b). The overexpression and knockout of *OsWOX10* respectively increase and decrease the densities of L-type lateral roots, showing that OsWOX10 positively regulates the formation of L-type lateral roots (Fig. 10B, 10C) (Kawai *et al.* 2022b). As QHB/OsWOX5 directly binds to the *OsWOX10* promoter and the transcription of *OsWOX10* is stimulated in the *qhb* mutant, in which a premature stop codon disrupts the conserved domains of WOX5 orthologues (Kawai *et al.* 2022b), QHB/OsWOX5 negatively regulates the *OsWOX10* expression to suppress the L-type lateral root formation (Fig. 10B, 10C) (Kawai *et al.* 2022b). The activator type OsARF19 binds to the promoter of *OsWOX10*, the expression of which is stimulated by the exogenous IAA treatment, indicating that OsARF19-mediated auxin signaling stimulates the L-type lateral root formation in rice (Fig. 10C) (Kawai *et al.* 2022a).

L-type lateral roots are abundant in the region proximal to the excision site in the seminal roots (Kawai *et al.* 2017), supporting that the auxin transport contributes to the balancing of two types of lateral root formations. Indeed, the convex side in the bending region of the crown roots, where auxin is highly accumulated, produces L-type lateral roots (Lucob-Agustin *et al.* 2020). The clathrin-mediated endocytosis of PIN proteins is required for the proper auxin distribution (Dhonukshe *et al.* 2007, Geldner *et al.* 2001). In *Arabidopsis*, large GTPase proteins, DYNAMIN-RELATED PROTEIN1A (DRP1A) and DRP2B are involved in the clathrin-coated vesicle formation (Fujimoto *et al.* 2010). Interestingly, the causal genes of rice mutants, in which L-type lateral roots increase, are *OsDRP1C* and *OsDRP2B* (Kawai *et al.* 2022a). In the seminal roots of the *drp* mutants with developing lateral root primordia, the *OsWOX10* expression, which is stimulated by auxin, is higher than the wild type (Kawai *et al.* 2022a), indicating that the clathrin-mediated endocytosis controls the PIN-mediated auxin distribution, thereby balancing the S-type and L-type lateral root formations (Fig. 10C).

Local auxin distribution is essential for the S-type and L-type lateral root formations. In the S-type lateral root primordia at the early stage, the signal of *DR5*::*NLS*-3x:*VENUS* is not detected, whereas, in the L-type, it is detected at the basal part of the crown root primordia (Kawai *et al.* 2022a). Although the Venus signal can be detected in the apical part of the S-type lateral root primordia, it is much stronger in the apical part of L-type lateral root primordia (Kawai *et al.* 2022a). Thus, the L-type lateral root initiation might require a greater auxin level at the basal part and its redistribution to the apical part when compared to the S-type lateral root initiation. It should be noted that the L-type lateral root primordia require more time to establish a more complex apical meristem structure (Kawai *et al.* 2022a), suggesting that the PIN-mediated local auxin distribution affects the periclinal and anticlinal cell divisions of lateral root primordia. In *Arabidopsis*, SHR and SCR function in periclinal cell divisions of lateral root primordia (Goh *et al.* 2016). It would be of interest to know the analogous functions of OsSHR and OsSCR during the S-type and L-type lateral root development in rice.

### Breeding of root traits for crop improvement

The breeding of root traits is often contrasted with the Green Revolution in which rice and wheat production is dramatically increased by introducing the semidwarf genotypes capable of responding to fertilizer application without lodging (Lynch 2007). The Green Revolution was based on the improvement of aboveground architecture to achieve maximum yield in fertile soils (Lynch 2007). However, the high-yielding genotypes are vulnerable to low fertility and abiotic stressors (Uga 2021). To sustain the food and energy demands under ongoing climate change, the second Green Revolution achieved by breeding root traits, especially for root system architecture that determines root distribution in soils, is urgently needed (Bailey-Serres *et al.* 2019, Lynch 2007, Uga 2021). In rice, hundreds of QTLs for root traits have been reported (Courtois *et al.* 2009). However, few of them have been demonstrated to contribute to crop performance under certain environmental constraints (Uga *et al.* 2015).

*DRO1* is detected on chromosome 9 in RILs derived from a cross between the upland cultivar ‘Kinandang Patong (KP)’ and the lowland cultivar ‘IR64’ differing in root growth angles (Uga *et al.* 2013). The introgression of the functional allele of *DRO1* from KP into IR64 (Dro1-NIL) doubles the maximum root depth under the upland conditions (Uga *et al.* 2013). The yield performance of Dro1-NIL is comparable with IR64 under upland field conditions without drought, whereas the Dro1-NIL maintains the percentage of filled grains and grain weights under mild and severe drought conditions in which the parameters of IR64 significantly reduce (Uga *et al.* 2013). *qSOR1* (*SOIL SURFACE ROOTING 1*) is detected on chromosome 7 in RILs derived from a cross between a lowland cultivar ‘Gemdjah Beton (GB)’ having soil surface roots and a lowland cultivar ‘Sasanishiki (SA)’ lacking soil-surface roots (Uga *et al.* 2012). Interestingly, SOR1 is classified as a different clade of IGT family protein from DRO1 (Kitomi *et al.* 2020). Introgression of the non-functional *sor1* from GB into SA (*qsor1*-NIL) results in an increase of soil-surface roots (Kitomi *et al.* 2020). Although *qsor1* does not affect the yield performance of SA in the control paddies, *qsor1*-NIL shows significantly higher yields than SA in saline paddies (Kitomi *et al.* 2020). These researches demonstrate that modification of the root system architecture through the control of root growth angles is a reliable breeding approach to improve crop tolerance to various abiotic stresses.

Increasing the density of L-type lateral roots, which have a well-developed vasculature with secondary branched lateral roots (Kawai *et al.* 2017), is an alternative approach to improve drought tolerance, especially for the rainfed lowland rice fields containing a hardpan that severely restricts the root growth into deep soil layer (Lucob-Agustin *et al.* 2021). Interestingly, the density of L-type lateral roots as well as the expression level of *OsWOX10*, which is an accelerator of L-type lateral roots, significantly increase under drought conditions in a lowland rice cultivar ‘Taichung 65’ without the excision of root tips (Kawai *et al.* 2022b). These results suggest that L-type lateral roots contribute to the drought adaptation of lowland rice cultivars. Among the chromosome segment substitution lines (CSSLs) derived from the cross between the cultivars ‘Nipponbare (NP)’ and ‘Kasalath (KA)’, CSSL47 shows greater L-type lateral root production and higher photosynthesis rates and yield performance than NP (Suralta *et al.* 2010). QTL analysis of the L-type lateral root number in the F_2_ mapping population of NP/CSSL47 identifies *qLLRN* on chromosome 12 (Niones *et al.* 2015). Although the causal gene for *qLLRN* remains unknown, the F_2_ lines having the KA allele of *qLLRN* increase L-type lateral root number by 5.8% and shoot dry weight by 39.5% than NP under the soil moisture fluctuation (Niones *et al.* 2015). Further genetic studies will progress our understanding of the importance of L-type lateral roots for crop tolerance to continuous and intermittent drought.

Root anatomical traits support the development of root system architectures with respect to the water, nutrients and oxygen allocation within each individual root and the balancing of the metabolic cost of soil exploration (Lynch 2019, Yamauchi *et al.* 2021a). The QTL of the stele transversal area (*qSTA*), which associates with the QTL of the xylem vessel area, is detected on chromosome 9 in the F_3_ mapping population of IR64 and KP (Uga *et al.* 2008). Although the KP allele of *Sta1* does not affect the shoot and root growths in the IR64 background under hydroponic culture (Uga *et al.* 2010), the stele transverse area is positively correlated with the long root systems in 59 rice accessions under rainfed upland conditions (Uga *et al.* 2009). When considering that KP has a larger stele transversal area (Uga *et al.* 2008), a large stele (xylem) transverse area might support the long and/or deep rooting systems. Interestingly, Sta1-NIL maintains higher leaf water potential and photosynthetic rate than IR64 at least in some growth conditions (Phoura *et al.* 2020). Another anatomical trait that contributes to drought tolerance is root cortical aerenchyma (Lynch 2018). In maize, RILs with a higher proportion of aerenchyma have greater yields than the lower-aerenchyma lines under drought (Zhu *et al.* 2010). The greater aerenchyma formation is associated with less root respiration and deeper rooting (Zhu *et al.* 2010), indicating that root aerenchyma formation restricts the metabolic cost for individual roots, thereby supporting the development of root system architectures in maize under drought.

In terms of tolerance to soil flooding, constitutive aerenchyma formation, which is observed only in wetland species, is particularly important (Mano and Nakazono 2021). QTL analysis of the constitutive aerenchyma formation in the F_2_ and backcross mapping population of maize × teosinte (*Zea* *nicaraguensis*; a wild relative of maize that adapts to the frequently flooded lowlands) identifies *Qaer1.06-1.07* on chromosome 1 (Mano and Omori 2007). Although the causal gene for the *Qaer1.06-1.07* has not been identified, the high capacity of constitutive aerenchyma formation is associated with the flooding tolerance of *Z. nicaraguensis* accessions (Mano and Omori 2013). Moreover, the introgression of *Qaer1.06-1.07* from *Z. nicaraguensis* improves the tolerance of a maize-inbred line Mi29 to the onset of oxygen-deficient conditions (Gong *et al.* 2019). Although it remains to be addressed, variation of aerenchyma formation in different rice cultivars and/or the wild relatives will provide new insight into future rice breeding of various abiotic-stress tolerances.

## Concluding Remarks

In this review, we summarized the current understanding of genetic and molecular mechanisms controlling rice plant development, especially by focusing on the shoot and root architectures. We have seen that many genes identified from developmental mutants played important roles in improving these architectures of rice. Importantly, many of these cases utilized alleles of weak effects with slight increases/decreases in gene expression or partial modification of protein functions. This tendency tells us two lessons: the value of weak alleles in breeding and the importance of basic knowledge of gene functions. Studies using strong or null alleles allow us to understand mechanisms underlying the regulation of certain traits and provide theoretical frameworks for breeding. With the high precision of genome editing techniques, it is now technically possible to create mutant alleles with a desired extent of effects (Endo *et al.* 2015, 2016, 2019, Mikami *et al.* 2015). Recently, another promising strategy to create a continuum of mutant alleles, from null to full functional alleles, by modifying the promoter of target genes with multiple guide RNAs was proposed (Rodriguez-Leal *et al.* 2017). These approaches would allow one to create the optimum allele for a given trait, which is difficult to be identified among rare natural variations (Rodriguez-Leal *et al.* 2017). In case that cis-regulatory elements exist far away from the gene of interest, especially in crop species with larger genomes and even in rice, the identification of these regulatory sequences will be important. Functional genomics with a powerful computational analysis successfully revealed the landscape of cis-regulatory elements at the genome scale (Marand *et al.* 2021). Integration of these approaches would widen the potential of crop breeding.

Importantly, these reverse genetic approaches rely on the knowledge of gene function in a tissue or organ of interest. Every single piece of knowledge on gene functions potentially provides valuable opportunities for breeding. In this sense, organs such as roots as well as anatomical and physiological traits in any tissue that had not experienced extensive selection have great potential to provide opportunities for crop improvement. In addition, the accumulating knowledge in rice domestication and breeding would also encourage the de novo domestication of wild species and the improvement of orphan crops (Khan *et al.* 2019, Varshney *et al.* 2012, Yu *et al.* 2021). This approach will be important to create stress- and disease-tolerant crops by utilizing wild relatives to overcome climate and environmental changes. In this respect, the exploration of new strategies for plant transformation is crucial (Shimizu-Sato *et al.* 2020). Continued efforts in elucidating basic molecular mechanisms and in characterizing genetic diversities will constitute the foundation of future breeding.

## Author Contribution Statement

All authors designed, wrote, and revised the manuscript, and approved the final version of the manuscript.
